# The nuclear transportation of CHRONO regulates the circadian rhythm

**DOI:** 10.1016/j.jbc.2024.107917

**Published:** 2024-10-24

**Authors:** Qin Zhou, Yunxia Su, Ruohan Wang, Zhiyuan Song, Honghua Ge, Ximing Qin

**Affiliations:** 1 Institute of Health Sciences and Technology, Anhui University, Hefei, Anhui Province, China; 2 Institutes of Physical Science and Information Technology, Anhui University, Hefei, Anhui Province, China

**Keywords:** circadian rhythm, CHRONO, transcription co-repressor, NLS, pegRNA/CRISPR

## Abstract

The pace of the endogenous circadian clock is important for organisms to maintain homeostasis. *CHRONO* has been shown to be a core component of the mammalian clock and has recently been implicated to function in several important physiological aspects. To function properly, CHRONO needs to enter the nucleus to repress transcription. We have previously shown that the N terminus of CHRONO is required for its nuclear entry. However, how CHRONO enters the nucleus and regulates the circadian clock remains unknown. Here, we report that a novel nonclassical nuclear localization signal in the N terminus of CHRONO is responsible for its nuclear entry. Multiple nuclear transporters are identified that facilitate the nuclear import of CHRONO. We show that the Arg63 is the critical amino acid of the nuclear localization signal. Using prime editing technology, we precisely edit the Arg63 to Ala at the genomic loci and demonstrate that this mutation prolongs the circadian period, which is similar to knockdown of *CHRONO*. By using the CHRONO KO and R63A mutant cells, we also investigated the changes in the cytoplasmic/nuclear distribution of BMAL1. We show that BMAL1 localizes more in the cytoplasm in the deficiency of CHRONO nuclear entry. These results provide a model for CHRONO nuclear entry using a network of importins involved in the regulation of the circadian period.

Circadian oscillators exist in almost every human cell (1). In individual cells, rhythmicity originates from a cell-autonomous circadian clock machinery, which consists of a set of core clock genes interlocked by transcription-translation feedback loops (2). BMAL1 and CLOCK form an asymmetric heterodimer *via* PAS domains and activate the transcription of genes including *PER* and *CRY* through binding to E-box motifs in their promoter regions (3). In turn, PER and CRY proteins form complexes that translocate to the nucleus to repress CLOCK/BMAL1-mediated transcription (4). There are other auxiliary loops involved in the existence of the circadian clock. In the auxiliary loops, REV-ERBs (encoded by *REV-ERB*α, *REV-ERB*β) and RORs (encoded by *RORa*, *ROR*b, and *ROR*c) are activated when the CLOCK/BMAL1 heterodimer binds to the upstream region (5, 6). REV-ERBs regulate the expression of the *BMAL*1 gene in a negative way, whereas RORs regulate it in a positive way (7). In addition, DBP and NFIL3 act as activator and inhibitor, respectively, of D-box-mediated transcription of genes such as *Per3* (8, 9).

These clock genes make up the circadian oscillator, which controls important rhythmic outputs such as the sleep/wake cycle, hormone secretion, and metabolic rhythms (10). In order to ensure the proper expression of clock and clock-controlled output genes, these clock component molecules need to function properly in the nucleus. Nuclear transportation is thus a key process during circadian cycles. Some core clock proteins such as BMAL1, REV-ERB, PER, and so on have been shown to contain the nuclear localization signal (NLS) that promotes their nuclear cytoplasmic transport (11, 12, 13). The CLOCK/BMAL1 complex enters the nucleus through a BMAL1-dependent shuttle, and the NLS located at the N terminus of the BMAL1 enables it to achieve the nuclear cytoplasmic shuttle (14). CRY is necessary for stable PER2 phosphorylation, as well as PER1, PER2, and CK1ε accumulation within the nucleus (15, 16). Overexpressed CRY1 proteins are almost completely localized in the nucleus, while CRY1^1-496^ (without the C terminus) and CRY1^muNLS^ (with the NLS mutation at the C terminus) exhibit a distinct cytoplasmic distribution (17). The C terminus of CRY2 contains an NLS through which importin α1, importin α3, and importin α7 can transport PER2/CRY2 into the nucleus (16). Lee *et al*. revealed that KPNB1 can be independent of importin α to mediate PER/CRY entry into the nucleus (18). Genome-scale SiRNA screens have identified that knockdown of multiple importins can significantly prolong the circadian rhythm (19). Another systematic RNAi screening assay has reported that a nonclassical nuclear import carrier TNPO1 modulates circadian rhythms through PER1 translocation (20).

Recent studies have suggested that *CHRONO* is one of the core clock genes besides classical clock genes (21, 22, 23). *Chrono* is widely expressed in many tissues and organs, and knocking out of *Chrono* would lengthen the circadian period in mice (21, 22, 24). Interestingly, according to the RNA-sequencing study using multiple tissues from Baboons, *CHRONO* was shown to be expressed cyclically in the highest number of tissues (52 out of 64 tissues) (25), indicating a significant physiological role of CHRONO. CHRONO was reported to associate with the C terminus of BMAL1 to disrupt the interaction between BMAL1 and CBP/P300, resulting in the dysfunction of BMAL1 activity (23, 26). The N-terminal and C-terminal domains of CHRONO are intrinsically disordered, while the middle helical domain of CHRONO interacts with BMAL1 (26). Previously, we discovered that the N-terminal domain of CHRONO is the key domain for its nuclear localization (26), so we hypothesized that an NLS may exist in the N terminus of CHRONO, and that its nuclear entry is important for maintaining an appropriate circadian period. As a transcriptional inhibitor, CHRONO has been found to play an important role in the nucleus, thus, we planned to study the detailed nuclear entry of CHRONO which is crucial for the circadian clock.

Here, we identified a novel noncanonical NLS in the N terminus of CHRONO that mediates its nuclear entry. Initiated with immunoprecipitation followed with mass spectrometry (IP-MS) analysis, we showed that CHRONO interacts with several transporters, such as CSE1L, KNPB2, and KPNA5. RNAi knockdown of these nuclear transporters affected the cellular localization of CHRONO and significantly prolonged the circadian period, with an effect similar to that of KO of *CHRONO* in a couple of cell lines. Using pegRNA/CRISPR technology (27), we successfully mutated the endogenous Arg63 to Ala and demonstrated that the critical role of the NLS in regulating the circadian period. We also found that CHRONO deficiency affects the cytoplasmic/nuclear localization of BMAL1. Our data suggest that CHRONO uses a network of importins for efficient nuclear import to function properly in the regulation the circadian rhythm.

## Results

### A novel NLS peptide is located in the N terminus of CHRONO

**Figure 1:**
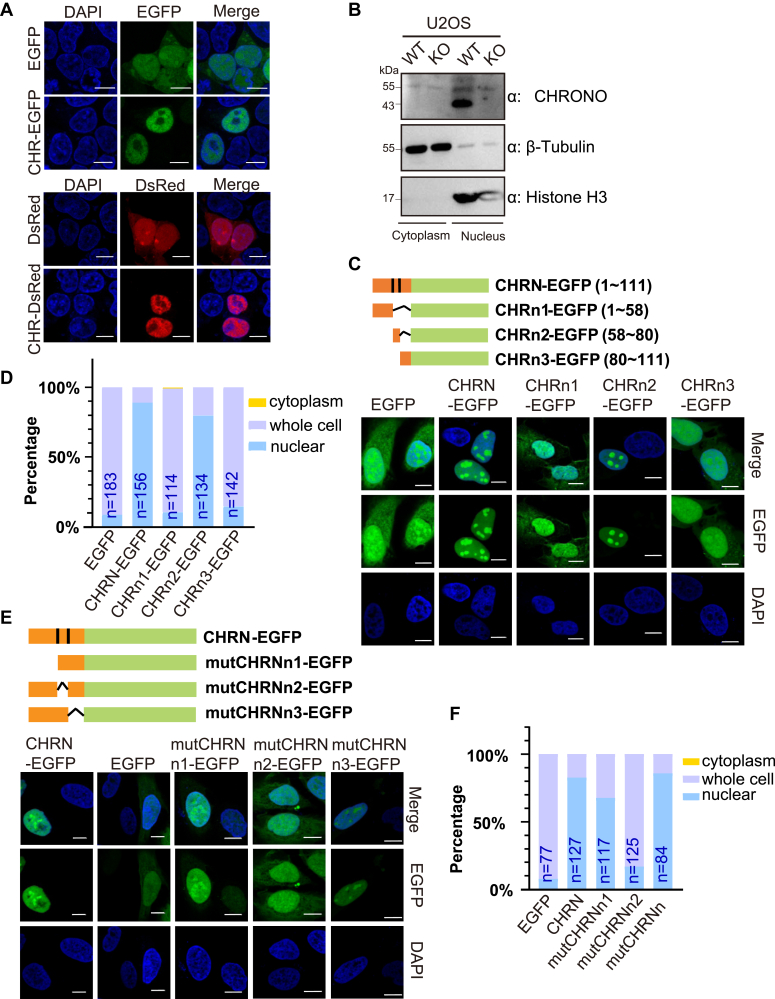
**The N terminus of CHRONO is essential for its nuclear localization.***A*, the fusion proteins CHR-EGFP and CHR-DsRed were expressed in 293T cells respectively, with EGFP and DsRed as controls. *B*, nuclear localization of endogenous CHRONO, using KO cell as a control. *C*, expression of different N-terminal truncated forms of CHRONO coupled to the fluorescent tag EGFP in U2OS cells, with EGFP as the control. The truncated length is labeled next to each construct. *D*, the proportion of cellular distribution of the coupled CHRONO in panel B was counted using ImageJ. *E*, expression of CHRONO with different mutations of the N segments in U2OS cells. *F*, localization statistics of panel D. Scale bar represents 10 μm. Experiments were carried out three independent times. The number of cells used for the statistics in panels *D* and *F* was shown.

Our previous study has shown that CHRONO mainly localizes to the nucleus to associate with BMAL1, and the N terminus of CHRONO leads to nuclear entry (26). Therefore, we made two different constructs consisting of CHRONO fused to fluorescent tags (CHR-EGFP, CHR-DsRed, and CHR is short for CHRONO) to further test the nuclear localization of CHRONO. Irrespective of the type of fluorescent tag, CHRONO is expressed mainly in the nuclei of 293T cells, whereas single EGFP or DsRed is expressed throughout the cells (Fig. 1*A*). Cellular fractionation assays confirmed that endogenous CHRONO is mainly localized in the nucleus (Fig. 1*B*). We found no classical NLS in CHRONO using online NLS prediction tools (28), but the ribosomal protein RPL28 clearly showed a predicted NLS between R112 and K135 of the protein sequence. Since functional regions of certain proteins have fewer single nucleotide polymorphisms across intraspecies (29), we analyzed the protein coding sequence of CHRONO using human data and found that the region of 50 to 80 amino acids is less variant (Fig. S1). In addition, most NLSs are short peptides rich in alkaline amino acids and contain the proline residue. Therefore, we divided the N terminus (CHRN, 1–111) of CHRONO into three smaller fragments: CHRn1 (1–58), CHRn2 (58–80), and CHRn3 (80–111). CHRn2 is rich in the alkaline amino acids, arginines, and lysines (Fig. S1). These fragments were fused to EGFP and transiently expressed in U2OS cells (Fig. 1*C*). After statistical analysis, the major nuclear localization region was found to be CHRn2 (58–80) (Fig. 1, *C* and *D*). To confirm that CHRn2 (58–80) has the NLS signal, different deletion mutations at the N terminus were constructed (Fig. 1*E*), complementary to those constructs shown in Fig. 1*B*. These constructs were transiently expressed in U2OS cells, and statistical analysis shows that deletion of the CHRn2 region results in a marked decrease in nuclear localization capacity (Fig. 1*F*).

### Arginine 63 is the core residue of the NLS

**Figure 2:**
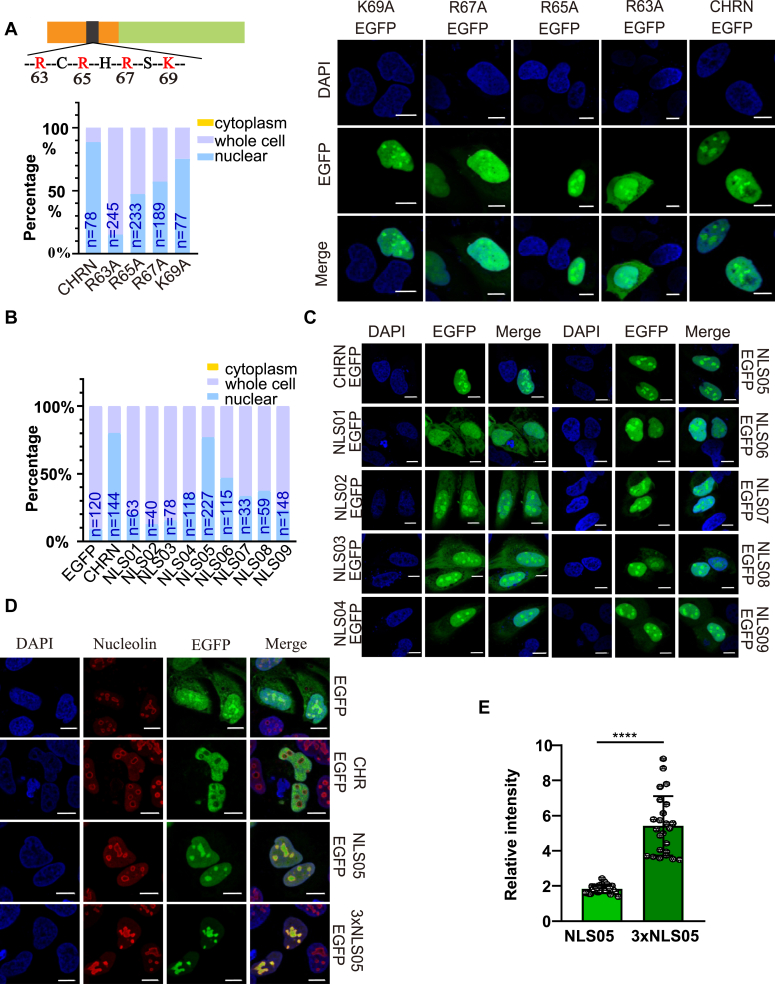
**A novel nuclear localization signal peptide is located in the N terminus of CHRONO.***A*, expression of different forms of the N terminus of CHRONO coupled to fluorescent EGFP with point mutations within the nuclear localization core sequence in U2OS cells. The images of the CHRN-EGFP are the same as those shown in panel *E* of Fig. 1. *B*, localization statistics of EGFP fused to nine different peptides that were derived from the nuclear localization segment (NLS) of CHRONO. The cellular distribution of the coupled EGFP in panel *C* was counted using ImageJ. *C*, localization of nine different NLS coupled with EGFP. EGFP fused with the N-terminus of CHRONO was used as a positive control. DAPI was used to stain the nuclei. *D*, NLS05 (as shown in Table S4) and 3 × NLS05 (concatenated in triplicate) coupled with EGFP (*green*) in U2OS cells. *Red* (Cy3 conjugated anti-nucleoin antibody) is marked as Nucleolin and *blue* (DAPI) is labeled as nucleus. *E*, efficiency of nuclear localization of NLS05 and 3 × -NLS05. Scale bar represents 10 μm. Experiments were carried out three independent times. The number of cells used for the statistics in panels *A* and *B* was shown. Error bars represent the mean ± S.D. (∗∗∗∗*p* < 0.0001). DAPI, 4′,6-diamidino-2-phenylindole.

As alkaline amino acids in classical NLSs are key residues for nuclear translocation, point mutations were performed on the four alkaline amino acids found in the CHRn2 region: R63A, R65A, R67A, and K69A, fused to EGFP and transiently expressed in U2OS cells (Fig. 2*A*). The results showed that CHRN lost its ability to enter the nucleus after the mutation of arginine at position 63 (Fig. 2*A*), indicating that the 63rd arginine is likely to be the core of the nuclear signal peptide. To determine the exact sequence of the NLS peptide, a series of small peptides fused to EGFP with the 63rd arginine as the core were constructed and expressed in U2OS cells (see Table S4 for amino acid sequence). Compared to the positive control that the N terminus of CHRONO mainly localizes in the nuclei, the peptide designated as NLS05 (59–71 of CHRONO, Fig. S1) can localize within the nucleus (Fig. 2*B*). When the peptide is either truncated or extended from both ends, the nuclear localization percentage dramatically decreases (Fig. 2, *B* and *C*). This signal peptide NLS05 was further concatenated in triplicate, named as 3 × NLS05, and we found that the signal peptide 3 × NLS05 is mainly located in the nucleolus (Fig. 2, *D* and *E*). We quantified the relative intensity of the green fluorescence by comparing the intensity in the nucleolus to that in the nucleoplasm. Subsequently, 3 × NLS05 significantly enhance the intensity in the nucleolus (Fig. 2*E*). Taken together, our results indicate that a noncanonical NLS is present in the N-terminal region of the CHRONO protein, and the 63rd arginine of the NLS is critical for its nuclear localization ability. Meanwhile, this novel signal peptide has nucleolar localization ability.

### CHRONO is translocated into the nucleus *via* multiple nuclear transporters

**Figure 3:**
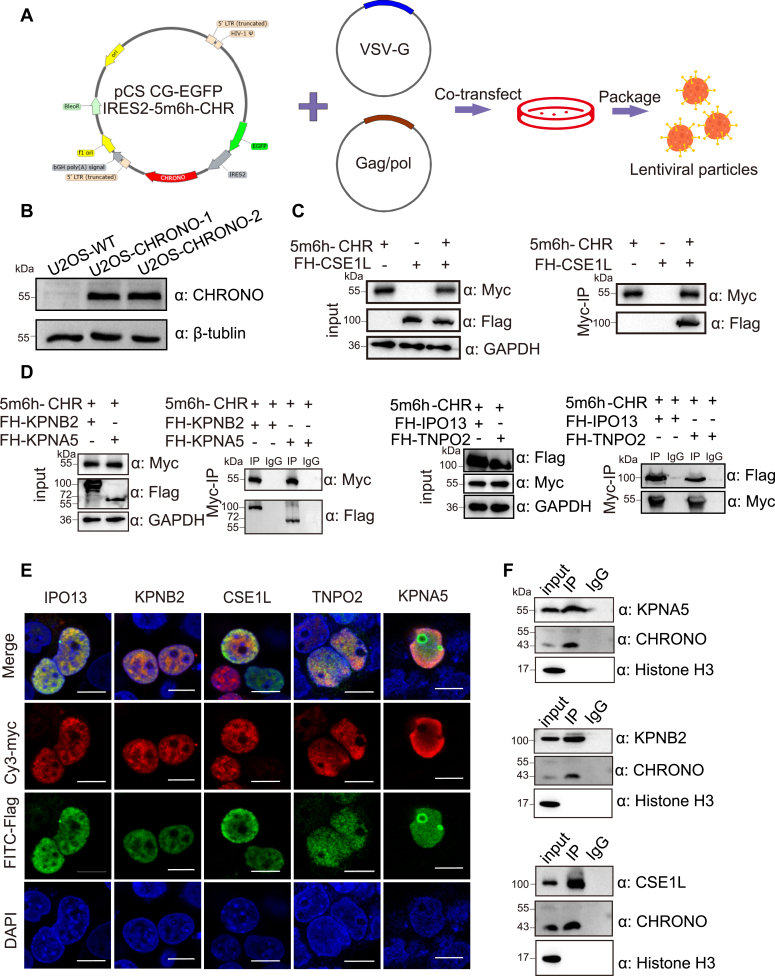
**CHRONO is translocated into the nucleus *via* multiple nuclear transporters.***A*, the strategy to establish the cell stably expressing CHRONO (CHR) using lentivirus. *B*, the antibody from Invitrogen (#PA5-55643) can recognize overexpressed 5 × Myc tagged CHRONO. *C*, 5Myc-6his(5m6h)-CHRONO, Flag-HA (FH)-CSE1L were cloned into pcDNA3.1 (+) vector, and transient transfection was performed in HEK293T cells. Whole cell lysates were immunoprecipitated with either anti-Myc antibody and WB analysis of the immunoprecipitation product was performed with either anti-Myc or anti-Flag antibodies, as indicated. GAPDH was used as the loading control for the input. *D*, 5m6h-CHRONO, Flag-HA labeled nuclear transporters were cloned into pcDNA3.1 (+) vector and the Co-IP assays were performed as indicated. *E*, immunofluorescence staining of CHRONO (cy3), nucleus (DAPI), and various nuclear transporters as indicated (FITC) in HEK293T cells. These images were the positive hits from the screening as shown in the Fig. S3*A*. Scale bar represents 10 μm. Experiments were carried out three independent times. *F*, endogenous CHRONO was detected in the precipitation products using antibodies to individual identified transporter. DAPI, 4′,6-diamidino-2-phenylindole; Co-IP, co-immunoprecipitation.

To understand the mechanism by which CHRONO enters the nucleus, two monoclonal cell lines U2OS-CHR-1 and U2OS-CHR-2 were constructed by stably expressing CHRONO using lentivirus (Fig. 3*A*). A 5Myc-6His tag was fused to the N terminus of CHRONO and we confirmed the stable expression of CHRONO using antibodies against the protein (Fig. 3*B*). We then screened the protein partners that may associate with CHRONO using IP-MS. Background controls used the same cellular extract using immunoglobulin G instead of the anti-Myc antibodies. Among the proteins interacting with CHRONO using IP-MS, we identified CSE1L, which belongs to the importin-β protein family (Fig. S2 for the list of identified proteins). We performed a co-immunoprecipitation (Co-IP) experiment where CSE1L was Flag-HA (FH-CSE1L) tagged and coexpressed with 5 × Myc-6 × His tagged CHRONO (5m6h-CHR). Consistent with the IP-MS results, CSE1L could be coimmunoprecipitated from cell lysates together with CHRONO proteins using an anti-Myc antibody (Fig. 3*C*). These data suggest that CSE1L helps CHRONO to enter the nucleus. We next asked whether other transporters are involved in the nuclear translocation of CHRONO. To this end, we screened the importin-α/β transporters (30) fused to the FLAG-HA (FH) tag using immunofluorescence (IF) colocalization and Co-IP assays (Fig. S3*A*). Consistently, CSE1L was coimmunoprecipitated with 5Myc tagged CHRONO proteins using an anti-Myc antibody (Fig. S3*C*). Among the importin-β family transporters, KPNB2, IPO13, and TNPO2 were found to coimmunoprecipitated with CHRONO in our screen (Fig. 3*D*). KPNA5 in the importin-α family also was coimmunoprecipitated with 5Myc tagged CHRONO protein (Fig. 3*D*). The IF images supported that the CHRONO protein is colocalized with KPNA5, KPNB2, CSE1L, IPO13, and TNPO2 (Fig. 3*E*). After reciprocal immunoprecipitation of Flag-tagged KPNA5, CSE1L, and KPNB2, Myc-labeled CHRONO was detected in the precipitation product, further supporting the association between CHRONO and these transporters (Fig. S4). We then isolated the nuclear extract from U2OS cells and precipitated KPNA5, KPNB2, and CSE1L individually. WB analysis demonstrated the presence of endogenous CHRONO in the precipitation product (Fig. 3*F*), indicating the interactions between endogenous CHRONO and nuclear transporters. We further analyzed the localization of KPNA5, KPNB2, and CSE1L individually over a 24-h period and found that the nuclear localization of these transporters is relatively unchanged within a circadian period (Fig. S5).

### The circadian period was significantly lengthened when *CHRONO* was knocked out using CRISPR technology

**Figure 4:**
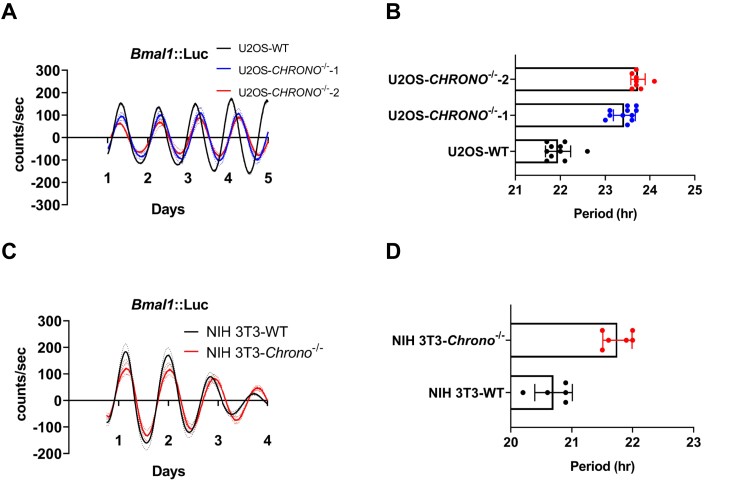
**KO of *CHRONO* significantly lengthen the period in different cell lines.***A*, two monoclonal CHRONO^−/−^ U2OS cell lines were generated using the CRISPR/Cas9 method. Representative subtracted bioluminescence data are shown for WT and *CHRONO*^−/−^ cell lines derived from U2OS cells expressing a *Bmal1*::Luc reporter. *B*, the period measured by the Lumi-Cycle luminometer is plotted for each cell line (n ≧ 6). The period of *CHRONO*^−/−^1 appeared to be prolonged by 1.48 ± 0.25 h, while *CHRONO*^−/−^2 was prolonged by 1.79 ± 0.16 h. *C*, a monoclonal *Chrono*^−/−^ NIH3T3 cell line was generated using the CRISPR/Cas9 method. Representative subtracted bioluminescence data are shown for WT and Chrono^−/−^ cell lines derived from NIH 3T3 cells expressing a *Bmal1*::Luc reporter. *D*, the period measured by the Lumi-Cycle luminometer is plotted for each cell line (n ≧ 5), and the period of Chrono^−/−^ cells appeared to be prolonged by 1.05 ± 0.24 h. Data are presented as the individual biological replicates or as an integration of all independent biological replicates (n = 2).

CRISPR technology has been successfully proved to be valuable for studying circadian rhythms (31). Using U2OS cells harboring a *Bmal1*::Luc reporter (32), we tested the effect of knocking out *CHRONO* gene using single guide RNA (sgRNA) targeting exon1 of *CHRONO* (Fig. S6*A*). Two monoclonal cell lines were successfully generated with indels close to the NGG site, resulting in early translation termination (Fig. S6*C* representing two alleles of *CHRONO*^−/−^1 cell line. For more details, see Supplementary Text S1). The KO of endogenous CHRONO in both cell lines was confirmed by Western blotting (WB) (Fig. S6*B*). Clearly, both CHRONO KO cell lines show significantly lengthened period compared to that of WT U2OS cells (1.48 ± 0.25 h and 1.79 ± 0.16 h longer, respectively, Fig. 4, *A* and *B*). In addition, we knocked out *Chrono* gene in NIH-3T3 cells harboring a *Bmal1*::Luc reporter (33). Compared to the luminescence trace of WT NIH-3T3 cells, the KO cells show a circadian period that is 1.05 ± 0.24 h longer than that of WT cells (Fig. 4, *C* and *D*).

### Knockdown of corresponding nuclear transporters also significantly lengthened the period

**Figure 5:**
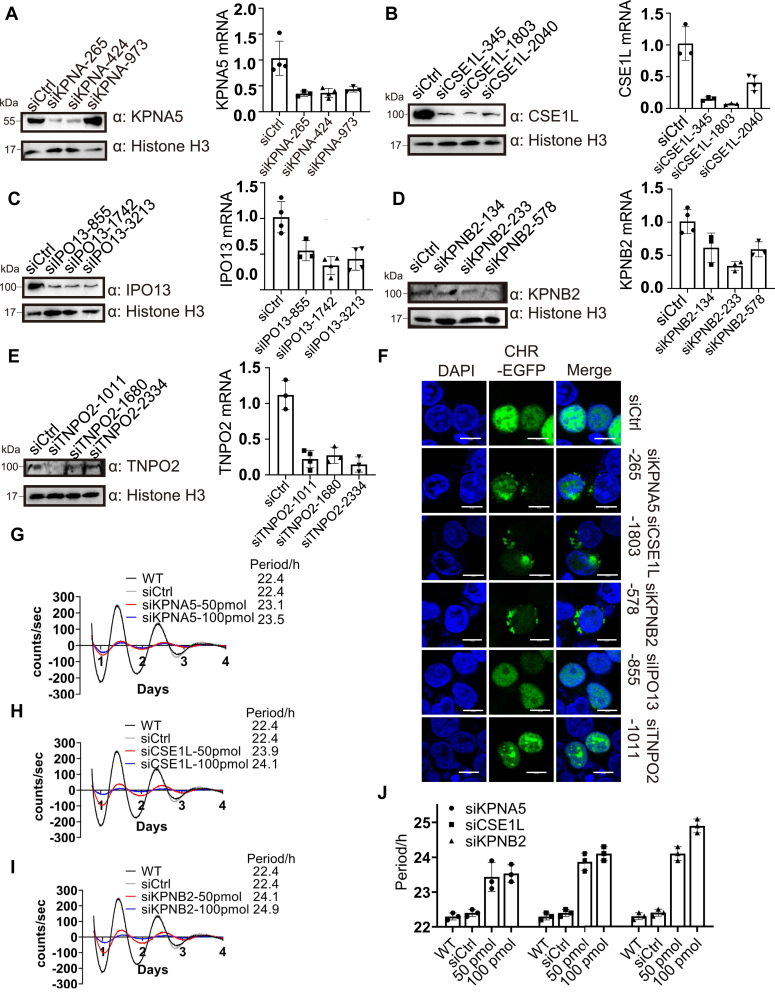
**Knockdown of corresponding nuclear transporters also significantly lengthened the period.***A*-*E*, three siRNAs were used for each transporter to test the efficiency of the knockdown at both protein and RNA levels. *F*, IF to show the effect of knockdown of individual nuclear transporters on the cellular localization of CHRONO. EGFP labeled CHRONO was cotransfected with siRNA that targets each nuclear transporter, and the fluorescence images were captured using a confocal microscopy. *G*, knockdown of KPNA5 by siRNA prolongs the period in a dose-dependent manner. *Black* = WT control. *Gray* = negative control siRNA. *Red* = 50 pmol siRNA. *Blue* = 100 pmol siRNA. *H*, knockdown of CSE1L significantly prolongs the period. *I*, knockdown of KPNB2 significantly prolongs the period. Scale bar represents 10 μm. Experiments were carried out three independent times. *J*, statistical analyses of the period when individual identified transporter was knocked down at different levels. Error bars represent the mean ± S.D. IF, immunofluorescence.

We then individually knocked down the transporters that could facilitate the nuclear entry of CHRONO using RNAi (sequences see Table S5). Initially, three siRNAs were used for each component we identified earlier to test the efficiency of the knockdown. According to the Western blot images and RT-PCR analysis, most siRNAs can dramatically knock down the transporters at both the protein level and the mRNA level (Fig. 5, *A*–*E*). We then selected the siRNA that knocked down each component the most to perform IF experiments. Individual SiRNA and CHR-EGFP were coexpressed in U2OS cells and fluorescent images were analyzed 24 h after transfection. We found that knockdown of KPNA5, CSE1L, and KPNB2 resulted in cytoplasmic localization of CHRONO, whereas knockdown of IPO13 and TNPO2 slightly affected the fluorescence intensity of GFP-tagged CHRONO (Fig. 5*F*). Thus, we chose KPNA5, KPNB2, and CSE1L to test the effect of their knockdown on the circadian clock. RNAi knockdown of KPNA5 slightly prolonged the circadian period (Fig. 5*G*), whereas RNAi depletion of the nuclear transporters CSE1L or KPNB2 significantly lengthened the circadian period in a dose-dependent manner (Fig. 5, *H* and *I*). Knockdown of KPNA5 lengthened the circadian period up to 1.1 h (Fig. 5, *G* and *J*), while knockdown of CSE1L and KPNB2 extended the period up to 1.7 and 2.5 h, respectively (Fig. 5, *H*–*J*). We also knocked down some transporters that we had not identified to interact with CHRONO in our screen, such as KPNA2 and IPO4 (Fig. S3, *B* and *C*). As we expected, knockdown of either KPNA2 or IPO4 did not affect the circadian period, or the localization of myc-tagged CHRONO proteins (Fig. S7).

### Prime editing of endogenous site that encodes the NLS of CHRONO

**Figure 6:**
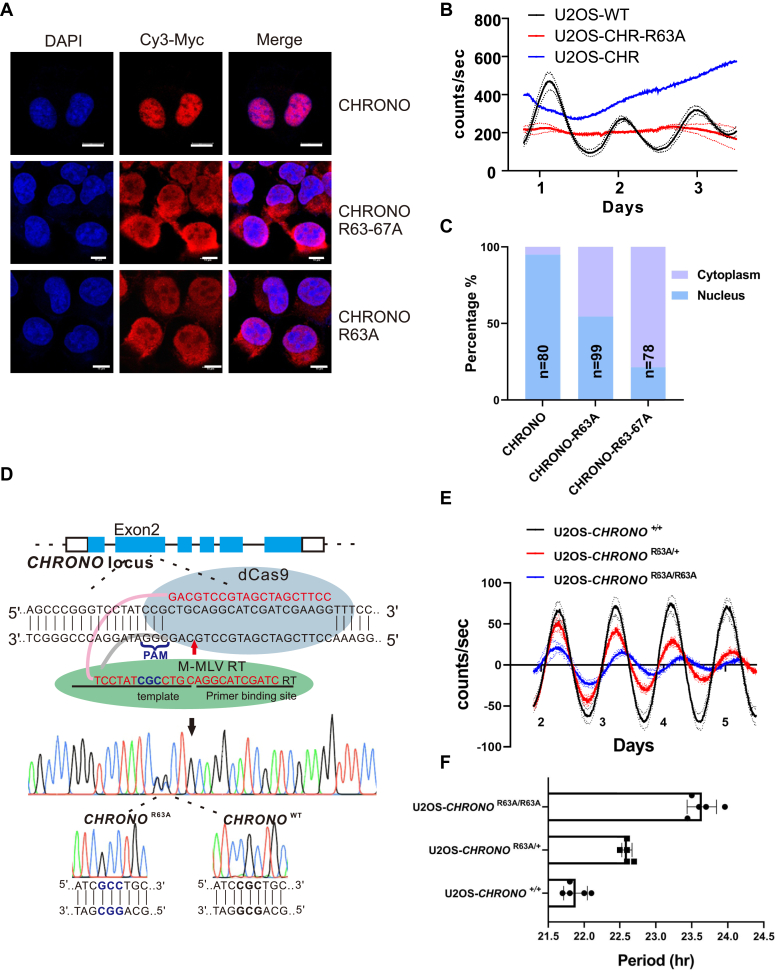
**Prime editing of endogenous site that encodes the nuclear localization signal of CHRONO.***A*, overexpressed CHRONO-R63A and CHRONO-R63 to 67A localized to both the cytoplasm and the nucleus. *B*, U2OS-CHR-R63A and U2OS-CHR-R63 to 67A cells were arrhythmic. *C*, quantification of the cytoplasm/nucleus localization of overexpressed CHR-R63A and CHR-R63 to 67A proteins. *D*, construction of the R63A mutation at the endogenous allele of *CHRONO* using pegRNA technology (prime editing). *E*, two single clone cell lines, U2OS-*CHRONO*^R63A/+^ and U2OS-*CHRONO*^R63A/R63A^, significantly affect the circadian period. *F*, quantification of the data in panel *E* shows the period lengthening effect in a gene dose-dependent manner. Experiments were carried out three independent times. Error bars represent the mean ± S.D.

Since the 63rd arginine of the NLS is critical for nuclear localization of CHRONO (Fig. 2*A*), we investigate how overexpression of WT and R63A-mutant of CHRONO affects the circadian clock. Overexpressed CHRONO-R63A and CHRONO-R63 to 67A localized to both the cytoplasm and the nucleus compared to the exclusive nuclear localization of overexpressed CHRONO (Fig. 6*A*). Compared to the U2OS-WT reporter cells, overexpression of CHRONO in U2OS cells dysregulates the circadian clock (Fig. 6*B*). Overexpression of CHRONO-R63A also disrupted the clock (Fig. 6*B*). Although R63A mutant reduced the nuclear portion of CHRONO proteins (Fig. 6*C*), significant amounts of CHRONO localized in the nucleus, which may lead to disrupted clock in the cells.

To test the function of NLS in normally oscillating cells, we attempted to introduce the R63A mutation into the endogenous allele of *CHRONO* using pegRNA/CRISPR technology (27). The pegRNA targeting the endogenous Arg63 site of *CHRONO* was designed and transfected into the U2OS-C26 cells (Fig. 6*D*). We successfully obtained two monoclonal cell lines: one with heterozygous mutation, named U2OS-*CHRONO*^R63A/+^, and the other one with homozygous mutations, named U2OS-*CHRONO*^R63A/R63A^ (Fig. 6*E*). As expected, both heterozygous and homozygous mutations in the endogenous Arg63 site prolonged the circadian period in a gene dose-dependent manner (Fig. 6, *E* and *F*). The circadian period of these two monoclonal cell lines was extended by 0.72 ± 0.07 and 1.66 ± 0.20 h, respectively. Therefore, CHRONO must be transported into the nuclei to properly regulate the circadian clock.

### CHRONO affects BMAL1 cytoplasmic/nuclear distribution

**Figure 7:**
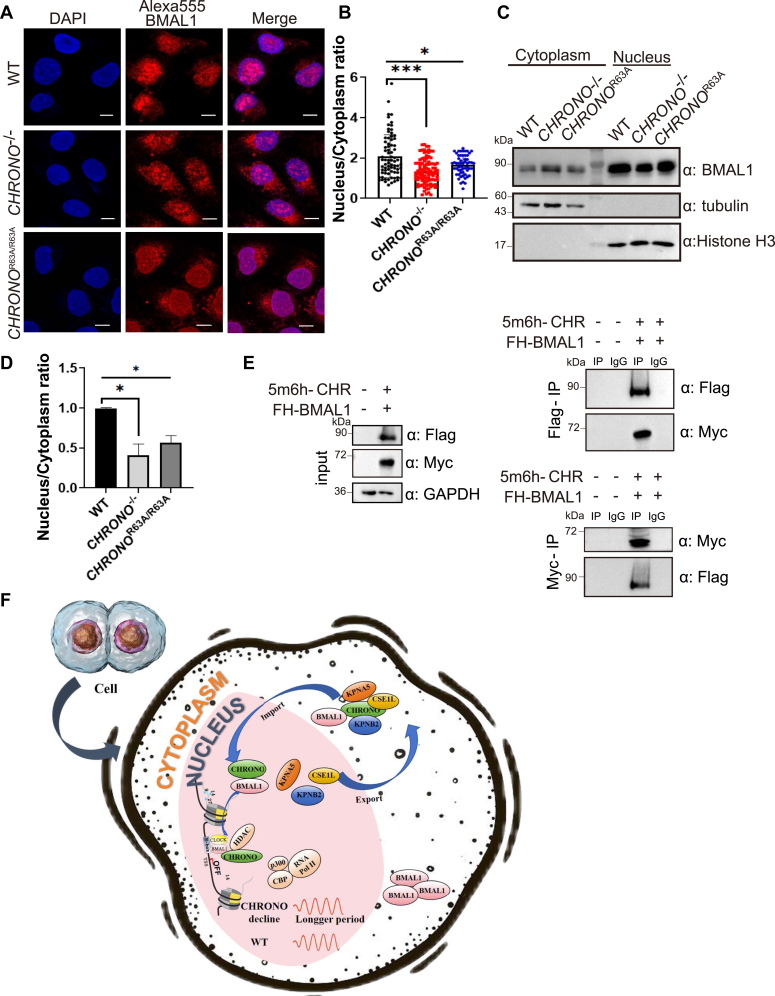
**CHRONO affects BMAL1 cytoplasmic/nuclear distribution and the model of CHRONO regulation of the circadian period.***A*, the cellular distribution of BMAL1 in WT, *CHRONO*^−/−^ and *CHRONO*^R63A/R63A^ cells was stained with Alexa555-conjugated antibody specific to BMAL1. Scale bar represents 10 μm. *B*, the nuclear and cytoplasmic intensities of Alexa555-BMAL1 were quantified and the nucleus to cytoplasmic ratio reflects the level of nuclear BMAL1. According to the statistics, BMAL1 localizes more in the cytoplasm than in the nucleus in the absence of CHRONO or endogenous R63A mutation. *C*, the cytoplasmic and nuclear fractions of both WT, *CHRONO*^−/−^ and *CHRONO*^R63A/R63A^ cells were separated and the distribution of BMAL1 was detected by immunoblotting. *D*, the nuclear and cytoplasmic immunoblotting band of BMAL1 was quantified and the nucleus to cytoplasmic ratio reflects the level of nuclear BMAL1. Error bars represent the mean ± S.D. n = 3(∗∗∗*p* < 0.001, ∗*p* < 0.05). *E*, Co-IP analysis to show the interactions between Flag labeled BMAL1 and Myc labeled CHRONO. *F*, the model of CHRONO entry into the nucleus which is involved in the regulation of the circadian period and the cytoplasmic/nuclear distribution of BMAL1. Co-IP, co-immunoprecipitation.

We used an IF assay to study the cellular distribution of BMAL1 in the WT, *CHRONO*^−/−^, and *CHRONO*^R63A/R63A^ cells (Fig. 7*A*). BMAL1 was detected using an Alexa555-conjugated antibody, and the nuclear and cytoplasmic fluorescence intensities were quantified using ImageJ (imagej.net/). In the absence of CHRONO, BMAL1 localizes more in the cytoplasm than in the nucleus (Fig. 7, *A* and *B*). When the critical arginine was mutated to alanine, BMAL1 also localizes more in the cytoplasm, as reflected by a lower nuclear ratio of the Alexa555 intensity (Fig. 7*B*). The changed distribution of BMAL1 was confirmed by immunoblotting when the cytoplasmic and nuclear fractions of these cells were separated. The immunoblotting bands of nuclear and cytoplasmic BMAL1 were quantified using ImageJ. Clearly, more BMAL1 protein was detected in the cytoplasm of *CHRONO*^−/−^ and *CHRONO*^R63A/R63A^ cells (Fig. 7, *C* and *D*). In our previous reports and other studies (22, 23, 26), CHRONO was reported to be tightly associated with BMAL1. Again, Co-IP experiments using antibodies to reciprocally precipitate the two proteins showed an obvious interaction between CHRONO and BMAL1 (Fig. 7*E*). Therefore, translocation of CHRONO influences the cytoplasmic/nuclear distribution of BMAL1.

## Discussion

In this study, we report several novel findings about the circadian component CHRONO, a transcriptional corepressor. First, we discovered a novel signal peptide located in the N-terminus of CHRONO that facilitates its nuclear entry. Second, multiple components of the importin α/β family proteins associate with CHRONO to translocate CHRONO into the nucleus. When these identified transporters were knocked down, the circadian period of U2OS cells were affected, which is similar to the effect of mutating the NLS at endogenous *CHRONO* loci. We also observed that knocking out *CHRONO* changed the cellular distribution of BMAL1, one of the core clock proteins. Taken together, our observations and discoveries highlight the role of CHRONO in regulating the period of the circadian clock.

Previously, we have found that the alpha-helical region of CHRONO interacts with BMAL1 to inhibit the transcriptional activation and have reported that the N terminus of CHRONO is required for its nuclear localization (26), but the NLS was not identified. In this report, a peptide with the sequence of PGPIRCRHRSKVS from CHRONO was found to be an NLS containing four basic amino acids (NLS05 in Fig. 2, *B* and *C*). This NLS sequence is found only in the CHRONO protein across different species. Characteristic of classical NLS is that the first position is lysine, followed by alkaline amino acids at the subsequent second and fourth positions, namely K(K/R)X(K/R) (34). For example, a classic single cluster NLS is the PKKKRRV of SV40 large T antigen (30), which is referred to as a single cluster NLS (34). The NLS of CHRONO contains four consecutive intervals of alkaline amino acids and two prolines in their vicinity with structural features close to the classical single cluster NLS (35). We therefore conclude that we have identified a novel NLS whose sequence characteristics differ from the classical single cluster NLS. Interestingly, while NLS05 and 3 × NLS-05 localize to the nucleolus, CHR-EGFP appears to be excluded from the nucleolus (Fig. 2). We speculate that the NLS alone may enter the nucleus together with some nucleolar localizing factors. When CHRONO enters the nucleus, the interactions between CHRONO and the nucleolar factor become weaker because CHRONO may interact with some other important component to perform its nuclear function.

Using IP-MS, we successfully observed CHRONO in the immunoprecipitation products. In addition, HNRNPDL (heterogeneous nuclear ribonucleoprotein D like) ranks first among the products (Fig. S2*A*). HRRNPDL is an RNA binding protein that has been found to be homogeneously distributed in both the nucleolus and the nucleoplasm (36). Importantly, CSE1L was observed in the protein list of IP products. It is an important protein in the classic nuclear transport pathway of cells. Using a Co-IP experiment, we have confirmed the interactions between CHRONO and CSE1L (Fig. 3*C*). We further used Co-IP to screen for other importin transporters that might interact with CHRONO, and several importin transporters were found to mediate the translocation of CHRONO into the nucleus.

It has been reported that multiple importin pathways serve as transporters for important proteins, such as core histones (37), ribosomal protein RPL23 (38), and c-JUN (39). Why proteins need multiple transporters and how they choose among different importins is largely unknown. In the case of CHRONO, it is difficult to decide which of the importins identified in this study defines the predominant one *in vivo*, since knockdown either of KPNA5, CSE1L, and KPNB2 can lead to partial cytoplasmic localization (Fig. 5*F*). Multiple importins of CHRONO may exist to ensure that CHRONO can be efficiently delivered to the nucleus to properly regulate the circadian clock (Fig. 5, *G*–*I*), indicating the importance of CHRONO. Unlike histones which contain at least two NLSs to interact with multiple importins (40), we found that CHRONO contains only one NLS (Fig. 2). CSE1L has been reported to be involved in the regulation of PER and CRY nuclear translocation (41). Also, KPNA5 is able to bind to CRY2 but not to an NLS mutant (16). In this study, CSE1L and KPNA5 were identified to interact with CHRONO for its nuclear entry. We speculate that CHRONO may form a large heterocomplex with these importins, together with other clock components, to enter the nucleus for circadian regulation. However, whether CHRONO, other clock proteins, and various importins form a large heterocomplex requires future studies.

Recently, CHRONO has been implicated in several functions. The gene expression levels of *CHRONO* and *BMAL1* have been shown to be used to discriminate individuals with bipolar disorders from normal controls (42). More interestingly, *CHRONO* was shown to control SARS-CoV-2 infection in both lung airway and lung alveolar organoids (43). Several core clock genes, such as *PER1*, *PER3*, *CRY1*, and *CRY2*, have been found to be associated with bipolar disorder (44). BMAL1 has been reported to regulate ACE2-dependent SARS-CoV-2 entry, as the ACE2 promoter encodes binding sites for the BMAL1/CLOCK complexes (45). CHRONO is known to negatively regulate the expression of genes that are activated by the BMAL1/CLOCK complexes through E-box elements (22, 23). Our findings in this study may provide more clues to understand the connection between CHRONO and these pathologies, further suggesting the importance of CHRONO.

Importantly, the cells need CHRONO to enter the nucleus in order to maintain their normal circadian period (Figs. 4 and 5, *G*–*I*). The NLS is critical for nuclear localization of CHONO. After mutation of the core amino acid residue, the nuclear cytoplasmic distribution of CHONO changes significantly, resulting in a decrease of CHONO in the nucleus and a significant prolongation of the rhythm. Here, we have further shown that loss of CHRONO or the R63 mutation can change the cellular distribution of BMAL1, resulting in more cytoplasmic BMAL1 (Fig. 7, *B* and *D*). As illustrated in the proposed model (Fig. 7*F*), multiple nuclear transporters help CHRONO to enter the nucleus. Since knockdown of IPO13 and TNPO2 only slightly affected the nuclear localization of CHRONO (Fig. 5*F*), these two transporters may be dispensable. There are some other possible components in this process, since the NLS alone localizes to the nucleolus. However, when CHRONO is intact, the association of CHRONO with other proteins, such as BMAL1, localize it to the genomic region responsible for gene regulation. Upon the nuclear entry, these transporters dissociate from CHRONO and are exported to the cytoplasm. Then CHRONO can compete with CBP/P300 to bind the C terminus of BMAL1 and subsequently recruit histone deacetylase to deacetylate histones to repress transcription. When CHRONO is knocked out or its nuclear transportation is blocked by mutation of the critical Arg63, the timing of transcriptional silencing is delayed, leading to a longer period of time. It is known that nuclear translocation of BMAL1 enhances proteolysis of both BMAL1 and CLOCK *via* ubiquitin-dependent and -independent pathways (14, 46). Further investigation will be necessary to test whether the changes of nuclear BMAL1 is also involved in the effect of longer period. As described in the model, the nuclear entry may be a point that can be manipulated to regulate the circadian clock.

Much evidence has suggested that misaligned rhythms can affect normal endocrine, metabolic and sleep/wake cycles, which could lead to metabolic disorders (47, 48), neuronal diseases (49), and aging problems (50, 51). Studies in animals have long suggested that the length of the endogenous clock is related to the phase angle after entrainment (52, 53). Organisms with short circadian period are entrained early and organisms with long circadian period are entrained late to the phase of the external environment, which is normally a 24-h cycle (54). Researchers have found that women’s circadian rhythms are shorter than men’s, which is consistent with the earlier phases of the rhythm marker melatonin and the sleep cycle in women (55, 56). These reports have provided strong evidence that an accurate circadian period is important for the adaptation to the environment. In this study, KO of *CHRONO* or mutations of the key residue of the NLS dramatically affect the circadian period. When the circadian period is altered, different environmental cues are required for the organism to be entrained. For example, additional light exposure is required to entrain humans to an environment longer than 24 h (57). In conclusion, CHRONO needs to function properly by entering the nucleus to maintain a normal circadian period.

### Limitations

This study has several limitations that need to be addressed in the future. First, U2OS cells were used in the localization assay and the liquid chromatography tandem mass spectrometry (LC-MS/MS) experiments. U2OS cells are a common model in the circadian field to study the cell physiology of circadian clocks. However, primary cells and more cell lines are required to verify the function of CHRONO proteins. Although we have knocked out *CHRONO* and its mouse homolog in a number of cell lines including U2OS and NIH3T3 cells to show the period effect of this gene deficiency, the possibility of off-target deletion or mutation of other genomic loci cannot be ruled out. We have sequenced the genomic region around the CHRONO loci, but unexpected mutations may need to be verified by using some high throughput sequencing methods, such as whole genome sequencing.

## Experimental procedures

### Immunofluorescence and localization

To visualize the expression of the protein in mammalian cells, CHRONO (CHR) was cloned into pcDNA3.1 with a C-terminal EGFP (CHR-EGFP) or DsRed (CHR-DsRed) tag. U2OS cells were grown in Dulbecco’s modified minimal Eagle’s medium (#C3113–0500, VivaCell) supplemented with 10% (vol/vol) fetal bovine serum (FBS, #FSP500, ExCell Bio), penicillin and streptomycin solution (#2233204, VivaCell) at 37 °C in a 5% CO_2_ humidified incubator. Subsequently, 1 × 10^5^ cells per well were seeded in a 6-well plate 1 day before transfection. When the cells reached 70%∼90% confluence, they were transfected with polyethylenimine (PEI, 1 mg/ml) with the following plasmids, respectively: 2 μg CHR-EGFP and 2 μg CHR-DsRed. Briefly, 48 h after transfection, cells were washed three times with ice-cold PBS and fixed in 4% paraformaldehyde for 30 min at room temperature. After three times PBS washes, cells were embedded in mounting medium containing 4′,6-diamidino-2-phenylindole (DAPI). Localization analyses and documentation were performed using IF microscopy (TCS SP8, Leica). For IF experiments, HEK293T cells were also grown under the same conditions as U2OS. Cells were transfected with PEI with various combinations of the following constructs (all in pcDNA3.1 background): Flag-HA-nuclear transporter (Table S1 and the primers to make constructs see Table S2), 5 × Myc-6 × His-CHR (5Myc6His-CHR), CHR-EGFP, 3 × NLS-EGFP. Briefly, 48 h after transfection, cells were washed three times with ice-cold PBS and fixed in 4% paraformaldehyde for 30 min at room temperature. The cells were then permeabilized and blocked with 0.5% TritonX-100 and 5% FBS in PBS for 1 h at room temperature, followed by sequential washes with PBS and incubated with Myc-tag antibody (#2276, Cell Signaling Technology, diluted in PBS containing 5% nonfat powder milk) or Flag-tag antibody (#14793, Cell Signaling Technology, diluted in PBS containing 5% nonfat powder milk) for 1 h at room temperature. After three 5-min PBS washes, cells were immunostained with Cy3-conjugated antibody (#D110082, BBI Biotech) or FITC-conjugated antibody (#D110155, BBI Biotech) for 30 min at room temperature. After the last three times PBS washes, the cells were embedded in mounting medium, antifading (with DAPI, S2110, Solarbio). The IF analyses and documentation were performed using IF microscopy (TCS SP8, Leica).

### *CHRONO* KO in U2OS and NIH-3T3 cells

*Chrono*^*−/−*^-*Bmal1*::Luc in NIH-3T3 and *CHRONO*^*−/−*^-*Bmal1*::Luc in U2OS cell lines were generated using the CRISPR/Cas9 system. The sgRNA1 with the highest quality score (CCGCTGCAGGCATCGATCGA) targeted to *CHRONO* (on-target locus: human Chr1: +150255862), sgRNA2 (CTGTCCCGGGGTCACCATGGCAG) targeted to *Chrono* (on-target locus: mouse Chr2: −131948868). The single-stranded sgRNA sequences designed for two candidate genes were annealed, phosphorylated, and ligated into the pX459 vector (pSpCas9{BB}-2A-Puro was a gift from Feng Zhang, Addgene plasmid No. 48139), which was digested by the *Bbs*I enzyme. The constructed plasmid of each individual gene was transfected into NIH-3T3-B1-B10 cells (33) or U2OS-C26 (32) cells according to the instructions of the ExFect Transfection Reagent (#T101, Vazyme Biotech). Twenty-four hours after transfection, cells were screened with 2 μg/ml puromycin. Next, monoclonal cell lines were screened using the limiting dilution method. After the selected monoclonal cell lines were grown to cell populations, we used the Genome Extraction Kit (#D1700, Solarbio) to extract their genomes. Primers (Chrono-KO-F/R and CHRONO-KO-F/R in Table S2) were designed to target ∼800 bp upstream and downstream of the target editing site to amplify the target fragment. According to the instructions of the T7E1 endonuclease kit (#E001, Viewsolid), the amplified target fragments were analyzed for gene editing results. Besides, the amplified target fragments were ligated into the pLB vector followed by subsequent sequencing to confirm the KO of the genes.

### *CHRONO* stably overexpressed in U2OS cells

*CHRONO* overexpressed cell lines in U2OS cells containing *Bmal1*::Luc (U2OS-CHR) were generated by infection with Lentivirus, as illustrated in Fig. 3*A*. The IRES2 were ligated into pCS-CG vector (Addgene plasmid No. 12154), which was digested with *Xho*I and *Xba*I enzymes. After obtaining pCS-CG-IRES2, 5Myc-6His-CHR was inserted using *Xba*I enzyme. The constructed plasmid was transfected into HEK293T cells together with pMD2.G (Addgene plasmid No. 12259) and psPAX2 (Addgene plasmid No. 12260) using PEI. Subsequently, 72 h after the transfection, the lentiviral particles were collected and concentrated with PEG8000. U2OS cells containing *Bmal1*::Luc (U2OS-C26) were infected with lentivirus carrying both GFP and 5Myc-6His-CHR. Overexpressing cells with GFP signals were selected for further experiments by fluorescence-activated cell sorting flow cytometry (MoFlo Astrios EQ, Beckman).

### Generation of *CHRONO*^R63A/+^ and *CHRONO*^R63A/R63A^ cell lines using prime editor 2 system

U2OS-C26 cells (2 × 10^5^) were seeded on 6-well plates (#10905–193, Corning). Twenty-four hours after seeding, cells were transfected at approximately 70% confluency with 10 μl PEI 40K (#40816ES03, Yeasen) according to the manufacturer’s protocols and 2.5 μg PE2-P2A-GFP plasmid (Addgene #132776), 2.5 μg pegRNA plasmid (constructed from pU6-pegRNA-GG-acceptor, Addgene #132777, the pegRNA sequence was 5′-CCTTCGATCGATGCCTGCAGGTTTTAGAGCTAGAAATAGCAAGTTAAAATAAGGCTAGTCCGTTATCAACTTGAAAAAGTGGCACCGAGTCGGTGCTCCTATCGCCTGCAGGCATCGATC-3′). After 3 days, cells were washed with PBS and dissociated with trypsin. Cells were then diluted with PBS supplemented with 5% (v/v) FBS and passed through a 40-μm cell strainer before sorting. Flow cytometry was carried out on CytoFLEX SRT (Beckman Coulter). Cells were treated with 3 nM DAPI 15 min before sorting. After gating for doublet exclusion, single DAPI-negative cells with GFP fluorescence above that of a GFP-negative control cell population were sorted into 96-well plate containing high glucose Dulbecco’s modified minimal Eagle’s medium with GlutaMax supplemented with 10% FBS. Cells were cultured for 15 days before genomic DNA extraction. Then the cell genome was used as template for PCR and sequencing (F: 5′-TTCCCGTAGGATCACCAGGTG-3’; R: 5′- TCTCCAGTTCACCATCTCTTGATC-3′).

### Western blotting and Co-IP

Rabbit antibody against Myc-tag (#2276, Cell Signaling Technology), mouse antibody against Flag-tag (#14793, Cell Signaling Technology), mouse antibody against GAPDH (60004-1-Ig, Proteintech), rabbit antibody against BMAL1 (#14020, Cell Signaling Technology), rabbit antibody against CHRONO (#PA5-55643, Invitrogen), KPNA5(#13963-1-AP, Proteintech), CSE1L (#22219-1-AP, Proteintech), IPO13 (#11696-2-AP, Proteintech), KPNB2 (#20679-1-AP, Proteintech), and TNPO2 (#17831-1-AP, Proteintech) were subjected to Western blotting according to the manufacturer’s protocol. For Western blotting, cells were harvested at the indicated time points after dexamethasone synchronization and lysed with the radio immunoprecipitation assay buffer (#P0013B, Beyotime) at 4 °C for 30 min, centrifuged at 12,000 rpm, and the soluble fraction was collected in a new tube for SDS-PAGE, followed by immunoblotting analysis with the specific antibodies.

For Co-IP, the desired plasmids were transfected into cells using PEI. After 48 h, cells were lysed in Western and IP cell lysis buffer (#P0013, Beyotime, Shanghai, China) at 4 °C for 30 min. The supernatant of the lysate was prepared after centrifugation at 4 °C, 12,000 rpm for 20 min and immunoprecipitated with mouse anti-Myc antibody or mouse anti-Flag antibody and mouse immunoglobulin G with gentle rotation at 4 °C overnight, then incubated with the Protein G magnetic beads (#9006S, Cell Signaling Technology) solution at 4 °C for 2 h. The beads were washed with pre-cold PBS for five times with gentle rotation. Samples were resuspended in the SDS loading dye, boiled for 10 min, and resolved by SDS-PAGE and followed by immunoblotting analysis with the specific antibodies.

### cDNA synthesis and q-PCR analysis

Total cellular RNA extraction was performed with Trizol reagent (#ET101, Transgene, Beijing, China) according to the manufacturer’s instructions. Next, complementary DNA (cDNA) was synthesized using the HiScript III RT SuperMix for quantitative PCR (qPCR) (+gDNA wiper) (#R323, Vazyme Biotech) and was subjected to qPCR using AceQ Universal SYBR qPCR Master Mix (#Q511–02, Vazyme Biotech) on a real-time fluorescence quantitative PCR instrument (Thermo Fisher Scientific, QuantStudio6 Flex). The value of each cDNA was calculated using the ΔΔCt method and normalized to the value of the house-keeping gene control *GAPDH*. Data were plotted as a fold of change. The sequences of primers were shown in Table S3.

### NLS prediction of the CHRONO protein

First, we accessed the HMM-based prediction method, NLStradamus, for predicting NLSs at: http://www.moseslab.csb.utoronto.ca/NLStradamus/ (28). Using the human ribosomal protein RPL28 as an example, the website predicted that the NLS of RPL28 is located between R112 and K135. However, the website cannot predict any NLS using the CHRONO protein sequence. Next, we started to chop the amino acids in the N terminus of CHRONO to find the part that is important for its nuclear localization.

### LC-MS/MS analyses

LC-MS analysis was performed by the Core Facility Center for Life Science of the University of Science and Technology of China. The 5Myc6His-CHRONO protein was stably expressed in U2OS cells, followed by immunoprecipitation with Protein G agarose beads which were incubated with anti-Myc specific antibody, and the samples were separated by SDS-PAGE. After Coomassie brilliant blue staining, the gels were soaked in 50 μl of pure water for 30 min, then removed and cut (1-2 mm particles). Then, 1 ml decolorizing solution (50 mM NH_4_HCO_3_: acetonitrile (ACN) = 1:1, V:V) was added, vortexed for 10 s, destained at 37 °C for 30 min, briefly centrifuged and dried; repeated several times until the blue faded. Then 500 μl of ACN was added to dehydrate the gel until it was completely white, the liquid was aspirated and the tube was opened to air dry, 10 mM DTT was added until the liquid covered the gel, then the gel was incubated at 56 °C for 1 h, after cooling to room temperature, the liquid was aspirated, 55 mM iodoacetamide was added rapidly until the liquid covered the gel, and the gel was left at room temperature in a dark room for 45 min. The liquid was then absorbed, washed twice with 500 μl destaining solution and once with pure water, 500 μl of ACN was added, vortexed for 5 min and finally air dried thoroughly. The gel was then completely covered with the 0.01 μg/μl trypsin and incubate overnight at 37 °C. The next day, 5x volume of 50% ACN was added, vortexed for 5 min, centrifuged at 5000*g* for 1 min, the supernatant was collected and repeated, finally the resulting supernatant was centrifuged at 25,000*g* for 5 min, and the supernatant was collected for lyophilization. Peptides were analyzed by LC-MS/MS using an EASY-nLC1000 system (Thermo Fisher Scientific) and a Q_Exactive plus mass spectrometer (Thermo Fisher Scientific). The mobile phase: A: 0.1% formic acid in water; B: 0.1% formic acid in 80% ACN; the gradients: 3 to 28% B in 70 min; 28 to 45% B in 35 min; 2 min to 90% B, and 10 min hold at 90%. Thermo Proteome Discoverer 2.2 (Thermo Fisher Scientific) was used for protein identification using the uniprot-homo database with the following parameters: Precursor ion mass tolerance: ± 10 ppm; fragment ion mass tolerance: ± 0.02 Da; missed cleavage site: 2; false discovery rate of all peptide and protein identifications <1%; max missed cleavages: 2; minimum peptide length: 6; maximum peptide length: 144.

### Bioluminescence recoding and data analysis

Briefly, 2 × 10^5^ U2OS-WT, U2OS-*CHRONO*^−/−^, NIH3T3-WT, NIH3T3-*Chrono*^−/−^ cells were seeded in 35 mm dishes the day before synchronization. On the next day, cells in each dish were synchronized with 2 h treatment of 200 nM dexamethasone (dissolved in dimethyl sulfoxide) and recorded in a LumiCycle as described previously (26). Bioluminescence data were analyzed with the LumiCycle analysis program (Actimetrics) to obtain circadian parameters such as period and amplitude.

### Statistical analysis

Data of the period length, fluorescence intensity, mRNA quantification, and quantification of WB images were presented as the mean ± SD. They were obtained from three (or more) independent experiments. An unpaired Student’s *t* test was applied to compare the mean between two independent groups. *p* < 0.05 was considered statistically significant. For multigroup comparison, *p* values were derived from one-way ANOVA with Tukey’s correction for multiple comparisons. ∗∗∗∗*p* < 0.0001, ∗∗∗*p* < 0.001, ∗∗*p* < 0.01, ∗*p* < 0.05. Data were analyzed using GraphPad Prism Version 6.01 software (https://www.graphpad.com/, San Diego).

## Data availability

The original datasets from the IP-MS experiments have been submitted to ProteomeXchange *via* the PRIDE database, the accession number is PXD046945. All other relevant data are available from the corresponding author on reasonable request.

## Supporting information

This article contains supporting information.

## Conflict of interest

The authors declare that they have no conflicts of interest with the contents of this article.
