# Supplementary material for: The nuclear transportation of CHRONO regulates the circadian rhythm
Source: J Biol Chem. 2024 Oct 24;300(12):107917. doi: 10.1016/j.jbc.2024.107917 (PMC11599456; doi:10.1016/j.jbc.2024.107917)
Supplement: Supplementary Figures [file mmc1.pdf]

Supplementary Figure Legends

Figure S1. Analysis of the amino acid composition and single nucleotide diversity of CHRONO.

(A) The amino acid sequence of CHRONO and the single nucleotide diversity analysis of CHRONO.

Figure S2. IP-MS to identify the potential interaction partners for CHRONO.

(A) The protein list that identified as the potential interaction partners for CHRONO using IP-MS/MS.

Figure S3. Screening of nuclear transporters associated with CHRONO nuclear entry.

(A) Subcellular localization of 5M6H-CHRONO fusion protein and FH-nuclear transporter protein. HEK293T cells were transfected with plasmid DNA expressing 5M6H-CHRONO protein and FH-nuclear transporter. 48 h post-transfection, cells were fixed and visualized by fluorescence microscopy Cy3-Myc, FITC-Flag and DAPI staining. Scale bar represents 10 μm. **(B-C)** Immunoblots showing expression of proteins in 293T cells transfected with the indicated plasmids after IP with anti-Myc antibody or IgG. Input indicates immunoblotting result of total cell lysates.

Figure S4. CHRONO can be precipitated using reciprocal antibodies to Flag tagged transporters.

CHRONO was co-expressed with Flag-tagged KPNA5, CSE1L, and KPNB2 individually and it can be detected in the precipitation products using the Flag antibody.

Figure S5. The cellular localization of KPNA5, KPNB2, and CSE1L individually over a 24-hour period.

(A-C) The immunofluorescence images of endogenous transporters over a circadian period. T0, T6, T12, T18 represent the time (hour) after the cells were synchronized. **(D)** Statistical analyses of the nuclear and cytoplasmic distribution of each endogenous transporter.

Figure S6. The strategy to obtain *CHRONO* knockout cells.

(A) *CHRONO*$^{-/-}$ cells were derived from U2OS using CRISPR/Cas9 technology, sgRNA was targeted at exon 1. **(B)** Western blotting result has shown that CHRONO were knocked out in both U2OS- *CHRONO*$^{-/-}$ cell lines. (**C**) The genome DNA and protein sequence of WT and *CHRONO*$^{-/-}$-1 cells were compared, and protein translation is prematurely terminated. The information about other *CHRONO* KO cells can be found in Supplementary Text S1.

Figure S7. Knockdown of either KPNA2 or IPO4 did not affect the circadian period.

KPNA2 and IPO4 were found not to interact with CHRONO and were therefore used as negative controls to examine the effect of SiRNA knockdown on the circadian period. Knockdown of either KPNA2 or IPO4 did not affect the circadian period, or the localization of myc-tagged CHRONO proteins.

Figure S1

A

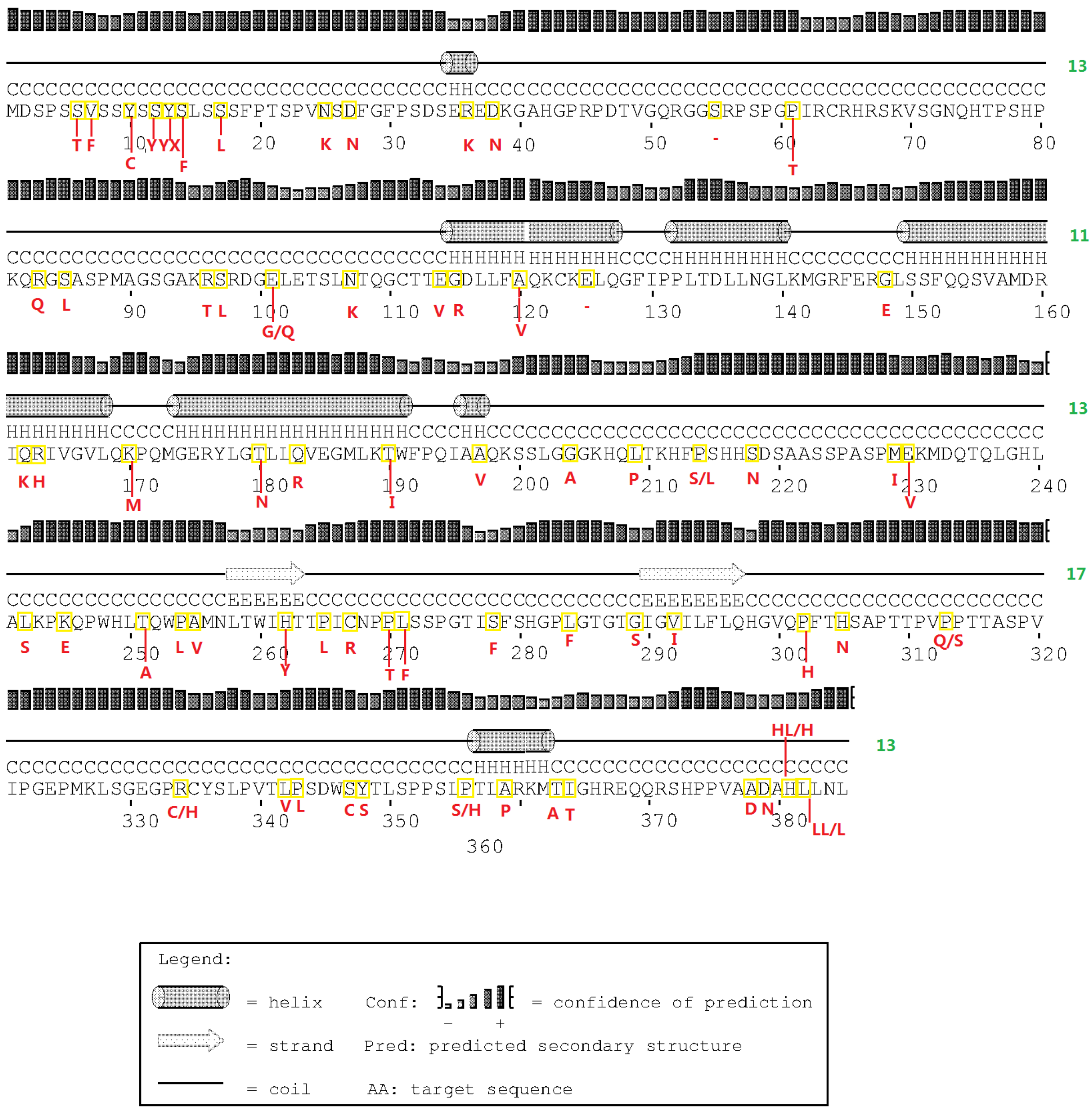

Total number of amino acids: 385

Total number of mutated amino acids: 67

Full-length mutation rate: 67/386 (0.174025974025974)

N-terminal mutation rate: 19/111 (0.171171171171171712)

Helix mutation rate(11-196): 11/86 (0.127906976744186

C-terminal mutation rate: 27/134 (0.2014925373134328)

Figure S2

A

Accession	Description	Sum PEP Score	Coverage [%]	# Peptides	# PSMs	# Unique Peptides	# AAs	MW [kDa]	calc. pI	Score Sequest HT:
O14979	Heterogeneous nuclear ribonucleoprotein D-like OS=Homo sapiens GN=HNRNPDL	21.745	21	4.5	11	6	420	46.4	9.57	31.83
Q8N365	Circadian-associated transcriptional repressor OS=Homo sapiens GN=CIART	20.464	29	8	9	7	385	41.4	9.42	28.47
O95816	BAG family molecular chaperone regulator 2 OS=Homo sapiens GN=BAG2	8.904	19	5	3	3	211	23.8	6.7	9.37
P23526	Adenosylhomocysteinase OS=Homo sapiens GN=AHCY	8.493	7	2	3	3	432	47.7	6.34	7.7
A6NHL2	Tubulin alpha chain-like 3 OS=Homo sapiens GN=TUBAL3	7.395	7	2	3	1	446	49.9	6.05	8.85
P27348	14-3-3 protein theta OS=Homo sapiens GN=YWHAQ	6.439	12	2.5	3	1	245	27.7	4.78	7.8
P51571	Translocon-associated protein subunit delta OS=Homo sapiens	5.787	14	1.5	2	2	173	19	6.15	4.91
P55060	Exportin-2 OS=Homo sapiens GN=CSE1L	5.146	7	3	5	5	971	110.3	5.77	3.83
P06730	Eukaryotic translation initiation factor 4E OS=Homo sapiens	3.624	5	1	1	1	217	25.1	6.15	2.99
Q9BW60	Elongation of very long chain fatty acids protein 1 OS=Homo sapiens GN=ELOVL1	2.788	5	1	1	1	279	32.6	9.6	2.43
Q92522	Histone H1x OS=Homo sapiens GN=H1FX	2.783	5	1	1	1	213	22.5	10.8	2.99
Q9UBX3	Mitochondrial dicarboxylate carrier OS=Homo sapiens GN=SLC25A10	2.605	8	1	1	1	287	31.3	9.54	3.22
P50990	T-complex protein 1 subunit theta OS=Homo sapiens GN=CCT8	2.444	2	1	1	1	548	59.6	5.6	3.14
P61619	Protein transport protein Sec61 subunit alpha isoform 1 OS=Homo sapiens GN=SEC61A1	2.085	2	1	1	1	476	52.2	8.06	2.63
P55884	Eukaryotic translation initiation factor 3 subunit B OS=Homo sapiens GN=EIF3B	2.002	1	1	1	1	814	92.4	5	2.44
P62937	Peptidyl-prolyl cis-trans isomerase A OS=Homo sapiens GN=PPIA	1.931	5	1	1	1	165	18	7.81	1.9
Q8WUF5	RelA-associated inhibitor OS=Homo sapiens GN=PPP1R13L	1.717	1	1	1	1	828	89	6.81	2.39
Q9Y3Z3	Deoxynucleoside triphosphate triphosphohydrolase SAMHD1 OS=Homo sapiens GN=SAMHD1	0.832	1	1	1	1	626	72.2	7.14	1.63

Figure S3

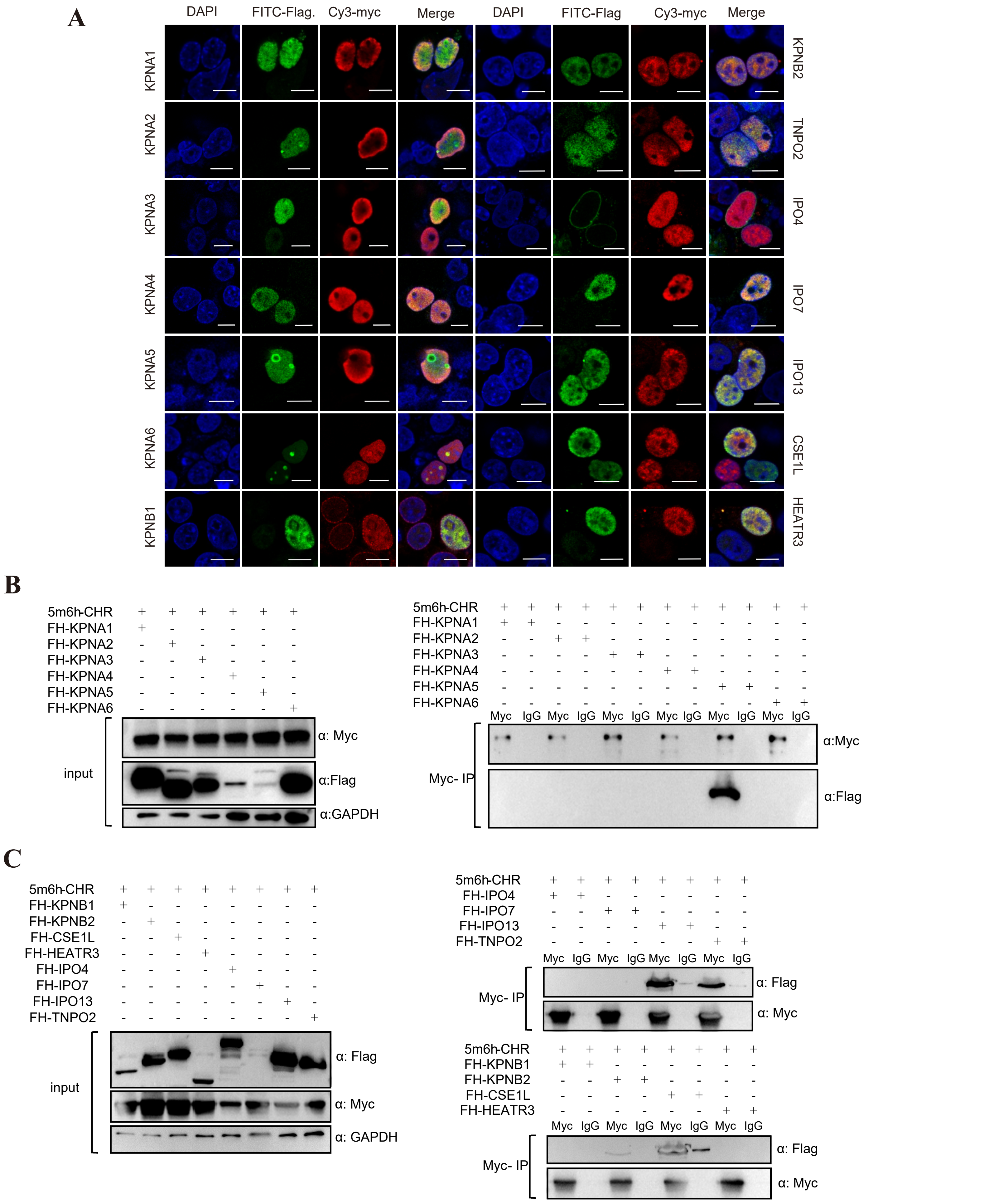

A
DAPI
FITC-Flag.
Cy3-myc
Merge
DAPI
FITC-Flag
Cy3-myc
Merge
KPNA1
KPNA2
KPNA3
KPNA4
KPNA5
KPNA6
KPNB1
KPNB2
TNPO2
IPO4
IPO7
IPO13
CSE1L
HEATR3
B
5m6h-CHR
FH-KPNA1
FH-KPNA2
FH-KPNA3
FH-KPNA4
FH-KPNA5
FH-KPNA6
input
α: Myc
α:Flag
α:GAPDH
Myc IgG Myc IgG Myc IgG Myc IgG Myc IgG Myc IgG
Myc- IP
α:Myc
α:Flag
C
5m6h-CHR
FH-KPNB1
FH-KPNB2
FH-CSE1L
FH-HEATR3
FH-IPO4
FH-IPO7
FH-IPO13
FH-TNPO2
input
α: Flag
α: Myc
α: GAPDH
Myc IgG Myc IgG Myc IgG Myc IgG
Myc- IP
α: Flag
α: Myc

Figure S4

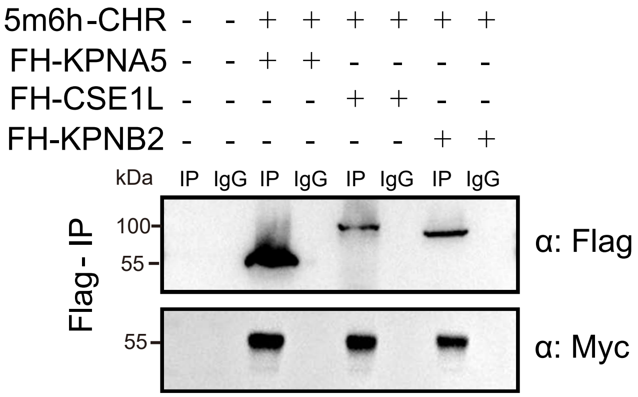

5m6h-CHR - - + + + + + +
FH-KPNA5 - - + + - - - -
FH-CSE1L - - - - + + - -
FH-KPNB2 - - - - - - + +
kDa
IP IgG IP IgG IP IgG IP IgG
Flag - IP
100
55
α: Flag
55
α: Myc

Figure S5

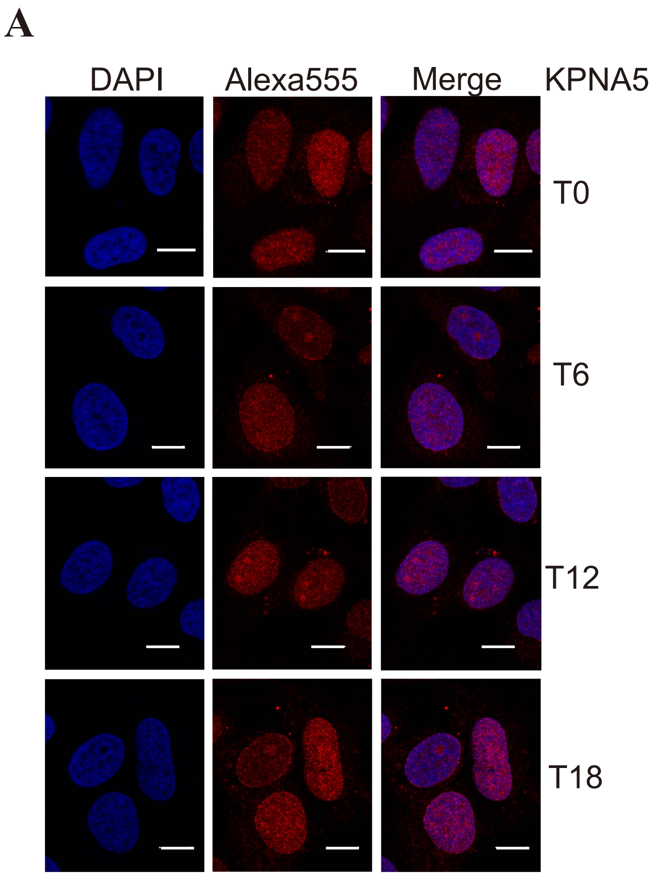
A
DAPI
Alexa555
Merge
KPNA5
T0
T6
T12
T18

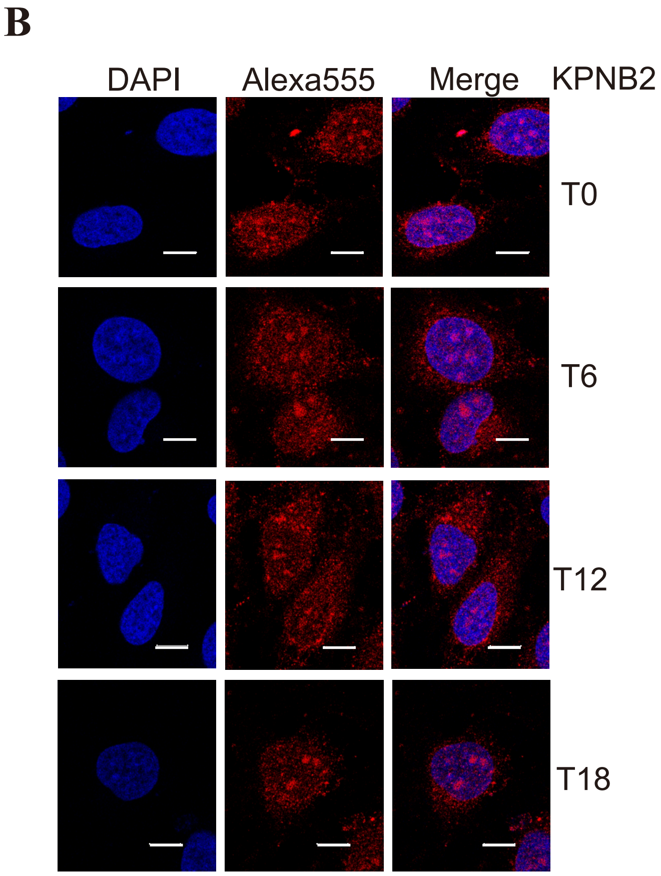
B
DAPI
Alexa555
Merge
KPNB2
T0
T6
T12
T18

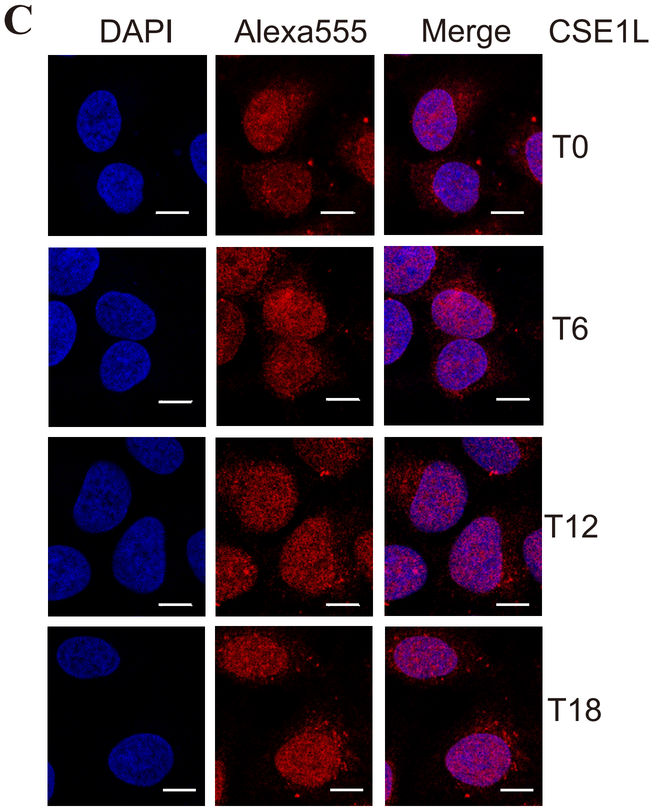
C
DAPI
Alexa555
Merge
CSE1L
T0
T6
T12
T18

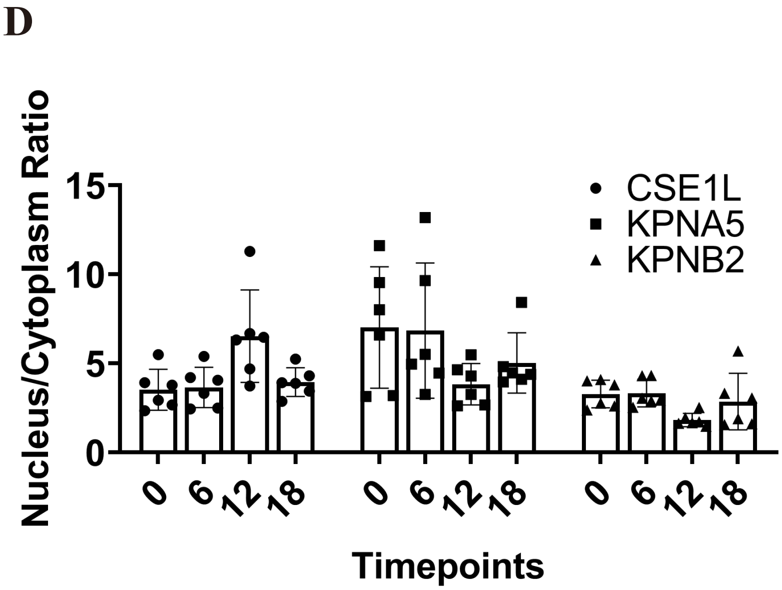
D
Nucleus/Cytoplasm Ratio
15
10
5
0
CSE1L
KPNA5
KPNB2
0
6
12
18
Timepoints

Figure S6

A

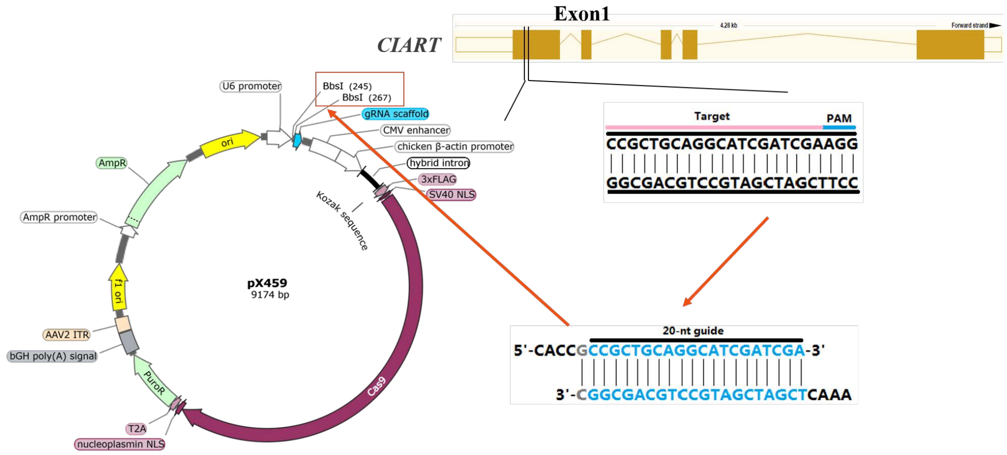

B

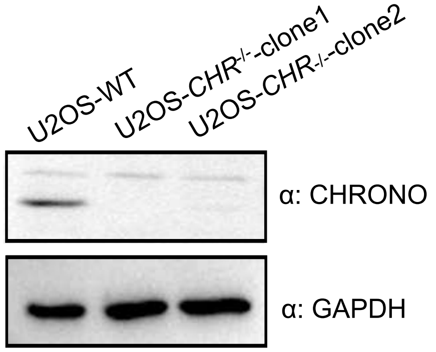

C

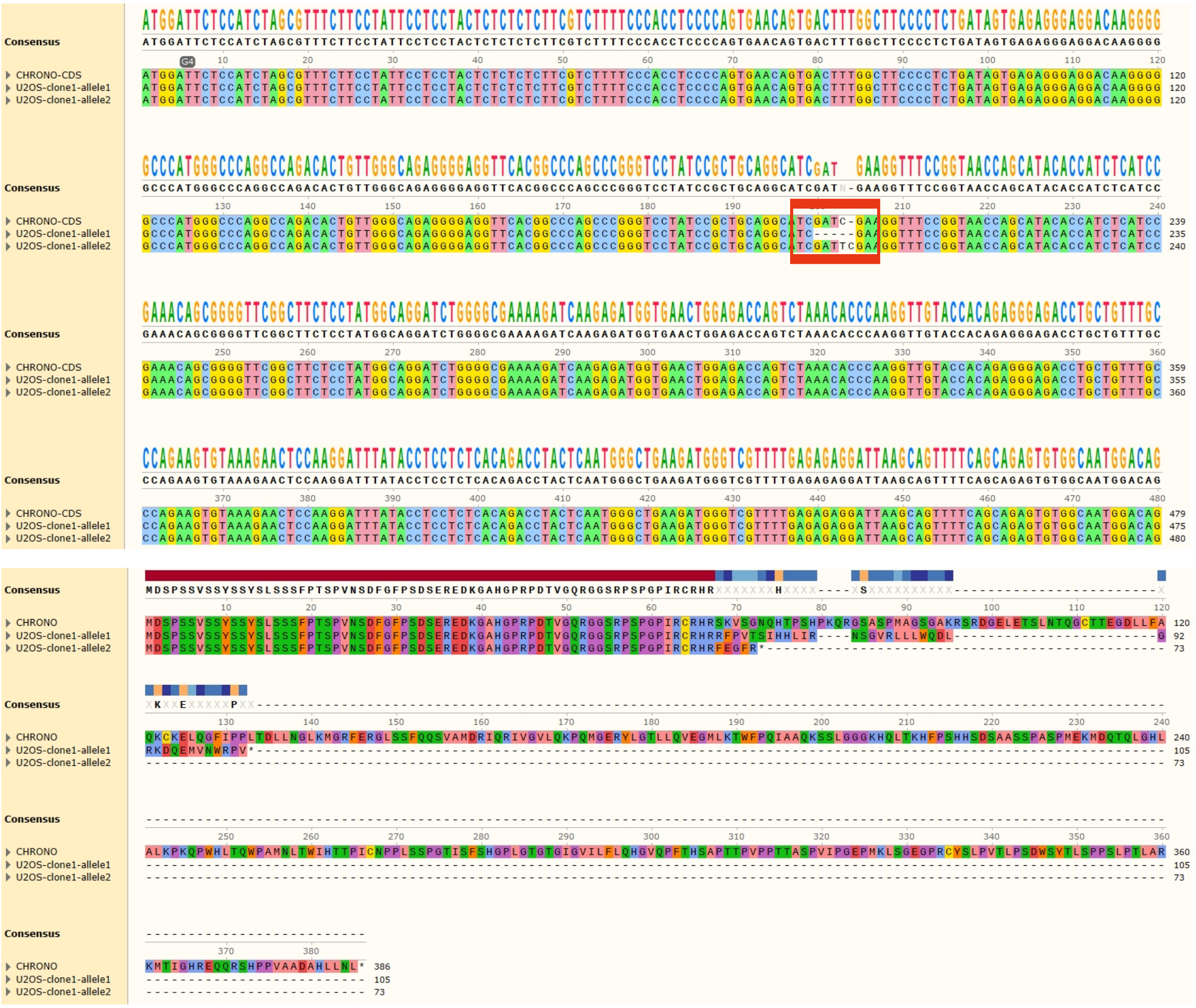

Figure S7

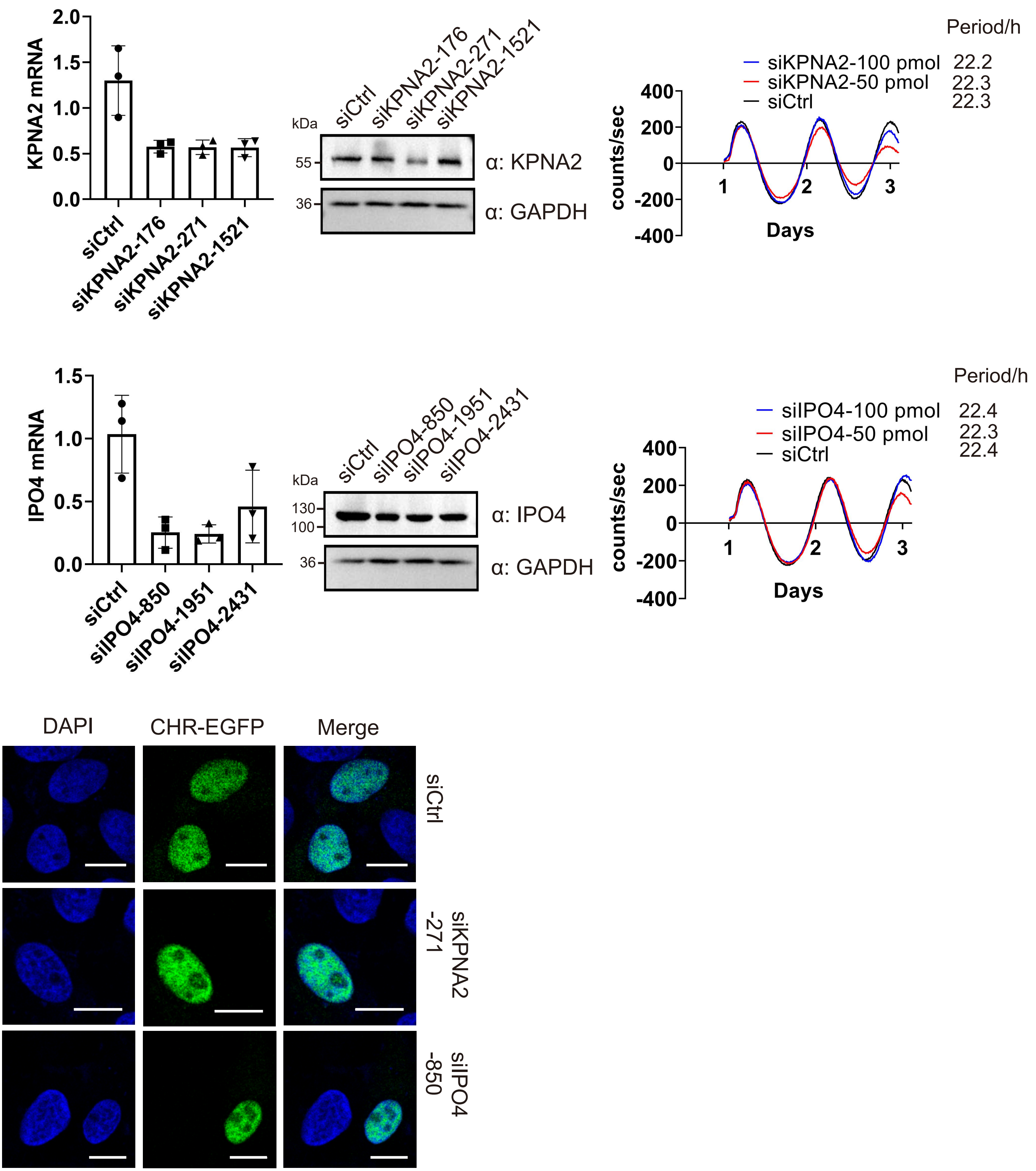

KPNA2 mRNA
2.0
1.5
1.0
0.5
0.0
siCtrl
siKPNA2-176
siKPNA2-271
siKPNA2-1521
kDa
55
36
α: KPNA2
α: GAPDH
Period/h
siKPNA2-100 pmol 22.2
siKPNA2-50 pmol 22.3
siCtrl 22.3
counts/sec
400
200
0
-200
-400
1
2
3
Days
IPO4 mRNA
1.5
1.0
0.5
0.0
siCtrl
siIPO4-850
siIPO4-1951
siIPO4-2431
kDa
130
100
36
α: IPO4
α: GAPDH
Period/h
siIPO4-100 pmol 22.4
siIPO4-50 pmol 22.3
siCtrl 22.4
counts/sec
Days
DAPI
CHR-EGFP
Merge
siCtrl
siKPNA2 -271
siIPO4 -850
