# Supplementary material for: The nuclear transportation of CHRONO regulates the circadian rhythm
Source: J Biol Chem. 2024 Oct 24;300(12):107917. doi: 10.1016/j.jbc.2024.107917 (PMC11599456; doi:10.1016/j.jbc.2024.107917)
Supplement: Supplementary Information [file mmc2.pdf]

The PDF File includes:

Table S1. Nuclear transporter list in the study

Table S2. Primers for cloning PCR

Table S3. Primers for RT-qPCR

Table S4. Sequences of signal peptide

Supplementary Text S1

Table S5. siRNA lists for knocking down nuclear transporters

Table S1. List of nuclear transporter in the study

Gene name	Study in the work
KPNA1	YES
KPNA2	YES
KPNA3	YES
KPNA4	YES
KPNA5	YES
KPNA6	YES
KPNB1	YES
KPNB2	YES
TNPO2	YES
TNPO3	Not study
IPO4	YES
IPO5	Not study
IPO7	YES
IPO8	Not study
IPO9	Not study
IPO13	YES
CSE1L	YES

Table S2. Primers for cloning PCR

Primer Name	Sequence (5'to3')
KPNA1-F	CCGGAATTCATGACCACCCCAGGAAAAGAGAAC
KPNA1-R	CCGGAATTCTCAAAGCTGGAAACCTTCCATAGG
KPNA2-F	CCGGAATTCATGTCCACCAACGAGAATGCTAATACACC
KPNA2-R	CCGGAATTCCTAAAAGTTAAAGGTCCCAGGAGCCCC
KPNA3-F	CCGGAATTCATGGCCGAGAACCCCAGCTTGGAGAACC
KPNA3-R	CCGGAATTCTTAAAAATTAAATTCTTTTGTTTGAAGG
KPNA4-F	CTACGCCGGAGGCGCGCCTGAATTCATGGCGGACAACGAGAAACTGGAC
KPNA4-R	CTGTGCTGGATATCTGCAGAATTCCTAAAACTGGAACCCTTC
KPNA5-F	CCGGAATTCATGGGGCGACAGCGGTCG
KPNA5-R	CCGGAATTCTTAAAGTTGAAATCCATCCATTGG
KPNA6-F	CCGGAATTCATGGAGACCATGGCGAGCCCAGGGAAAG
KPNA6-R	CCGGAATTCTTATAGCTGGAAGCCCTCCATGGG
KPNB1-F	CTACGCCGGAGGCGCGCCT GAATTCATGAAGGAGTCG ACATTGGAAG
KPNB1-R	CTGTGCTGGATATCTGCAGAATTCTCAAGCTTGGTTCTTCAG
KPNB2-F	CTACGCCGGAGGCGCGCCTGAATTCATGGAGTATGAGTGGAAACC
KPNB2-R	CTGTGCTGGATATCTGCAGAATTCTTAAACACCATAAAAAGCTGC
CSE1L-F	CTACGCCGGAGGCGCGCCTGAATTCATGGAACTCAGCGATGCAAATC
CSE1L-R	CTGTGCTGGATATCTGCAGAATTCTTAAAGCAGTGTCACACTGGC
IPO7-F	CTACGCCGGAGGCGCGCCTGAATTCATGGACCCCAACACCATTATCG
IPO7-R	CTGTGCTGGATATCTGCAGAATTCTCAATTCATCCCTGGTGC
IPO13-F	CCGGAATTCATGGAGCGGCGGGAGGAGCAG
IPO13-R	CCGGAATTCTCAGTAGTCAGCTGTGTAATCTGTG
IPO4-F	CTACGCCGGAGGCGCGCCTGAATTCATGGAGTCAGCCGGGCTAG
IPO4-R	CTGTGCTGGATATCTGCAGAATTCCTAGGAGAGGCCCAGTACAGCC
TNPO2-F	CCGGAATTCATGGACTGGCAGCCAGACGAGCAG
TNPO2-R	CCGGAATTCCTAGACCCCATAGAAAGCCGCCAGC
Chrono-KO-F	GGACAGGCGGACATAGGATG
Chrono-KO-R	ACACACCTGTTGCACAAACG
CHRONO-KO-F	ATGGATTCTCCATCTAGCG
CHRONO-KO-R	CTTCTGGGCAAACAGCAGGTC

Table S3. Primers for RT-qPCR

Gene name	Forward primer (5'to 3')	Reverse primer (5'to 3')
PER1	CATGAGTCTAGAGGCGCATC	TTGCTGCTCTCAGTGGTCTC
PER2	GCAAAATCTGAACACAACCC	CTTTGTGTGTGTCCACTTTC
PER3	TGTCTGTCCTCTGTTGTCGC	GCTGACGACATTGAGGGTGA
CRY1	TGATTCGTGGACAACCAGCAG	CCAAAGGGCTCAGAATCATAC
CRY2	GATCAACCGATGGAGGTTCC	TGTCCCCGGACTACAAACAG
BMAL1	ACATGCAACGCAATGTCCAG	TCTGTGTATGGATTGGTGGC
CLOCK	GCACTGTTGAAGAACCCAATG	TATTATGGGTGGTGCCCTGTG
NR1D1	TGCAGGCTGATTCCCCCTACA	AGCCCTGCAGAAGGGTTGGA
NR1D2	CACTATGGAGTTCATGCTTGC	CTTCAGGCACTTCTTGTACTG
RORα	CCTGCCAATACTTGAGAGAAG	CGCTGCTTGTTTTGATAGTTC
CIART	AGCATCAGCTGACCAAGCAT	TAGCTGGCCATTGTGTGAGG
DEC1	CCAGTCATCCAGCGGACTTT	GGGGGTTCTTCGGACTCTTG
DEC2	GCATGAAACGAGACGACACC	CCAGATGTCCCAGAGTTGTCA
E4BP4	GAGCCAAGAGATGACCGAGG	TGGAGGATCGGTTGACTTGC
DBP	CACCGTGGAGGTGTTGATGA	GAGGGTCAAAGGTCTCGTGG
RPLP0	ACGGGTACAAACGAGTCCTG	GCCTTGACCTTTTCAGCAAG
GAPDH	GTCTCCTCTGACTTCAACAGCG	ACCACCCTGTTGCTGTAGCCAA

Table S4. Sequence of signal peptide

Name	Signal peptide sequence
NLS01	IRCRHRS
NLS02	IRCRHRSKV
NLS03	IRCRHRSKVS
NLS04	PGPIRCRHRSKV
NLS05	PGPIRCRHRSKVS
NLS06	PGPIRCRHRSKVSG
NLS07	PGPIRCRHRSKVSGN
NLS08	PGPIRCRHRSKVSGN
NLS09	QPSPGPIRCRHRSKV

Supplementary Text S1

cDNA analysis of CHRONO transcript and protein predictions in various *CHRONO* knockout cell lines

To confirm that CHRONO is knocked out in the U2OS cell in this study, we have PCR and amplified the genomic region of the sgRNA target site (Fig.S4A) and confirmed the different in-dels of both alleles from U2OS-*CHRONO*$^{-/-}$-1 cells (Fig.S4C) as an example. In addition, to ensure that the corresponding *CHRONO* locus can be transcribed correctly, we isolated mRNA from each monoclonal cell line and reverse transcribed it into cDNA libraries. Using primers capable of amplifying the coding regions (CDS) of *CHRONO*, we successfully amplified the complete CDS from U2OS-WT, U2OS-*CHRONO*$^{-/-}$-1 (allele 1: -ATCG, -4bp; allele 2: +T, +1bp), U2OS-*CHRONO*$^{-/-}$-2 (allele 1: -AGGCATCGATCGAAGGTTTCCGGTAACC, -28bp; allele 2: -TCGAAGGTTTCCGGTA, -16bp). The amplicon size of *CHRONO* cDNA is 1155 bp. These CDS sequences were sent for complete sequencing and subsequent alignment confirmed that these loci can be correctly transcribed and that these loci contain only in-del mutations close to the sgRNA target site in exon-1 of *CHRONO*. As these CDS sequences are complete, including the start codon ATG and the stop codon TAG, we performed a protein prediction analysis. Our analysis showed that all these transcripts cannot be translated into correct CHRONO proteins, with very early translation stops caused by the in-dels. The alignments of these complete CDSs and the corresponding predicted proteins are shown below.

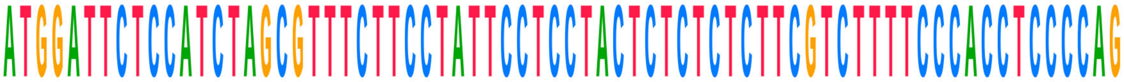

```
Consensus           ATGGATTCTCCATCTAGCGTTTCTTCCTATTCCTCCTACTCTCTCTCTTCGTCTTTTCCCACCTCCCCAG
                             10        20        30        40        50        60        70
CHRONO-CDS          ATGGATTCTCCATCTAGCGTTTCTTCCTATTCCTCCTACTCTCTCTCTTCGTCTTTTCCCACCTCCCCAG  70
U2OS-clone1-allele1 ATGGATTCTCCATCTAGCGTTTCTTCCTATTCCTCCTACTCTCTCTCTTCGTCTTTTCCCACCTCCCCAG  70
U2OS-clone1-allele2 ATGGATTCTCCATCTAGCGTTTCTTCCTATTCCTCCTACTCTCTCTCTTCGTCTTTTCCCACCTCCCCAG  70
U2OS-clone2-allele1 ATGGATTCTCCATCTAGCGTTTCTTCCTATTCCTCCTACTCTCTCTCTTCGTCTTTTCCCACCTCCCCAG  70
U2OS-clone2-allele2 ATGGATTCTCCATCTAGCGTTTCTTCCTATTCCTCCTACTCTCTCTCTTCGTCTTTTCCCACCTCCCCAG  70
```

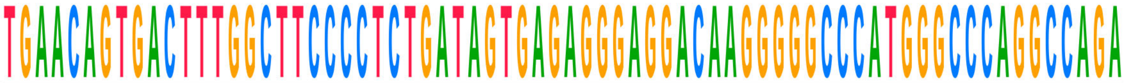

```
Consensus           TGAACAGTGACTTTGGCTTCCCCTCTGATAGTGAGAGGGAGGACAAGGGGGCCCATGGGCCCAGGCCAGA
                             80        90       100       110       120       130       140
CHRONO-CDS          TGAACAGTGACTTTGGCTTCCCCTCTGATAGTGAGAGGGAGGACAAGGGGGCCCATGGGCCCAGGCCAGA  140
U2OS-clone1-allele1 TGAACAGTGACTTTGGCTTCCCCTCTGATAGTGAGAGGGAGGACAAGGGGGCCCATGGGCCCAGGCCAGA  140
U2OS-clone1-allele2 TGAACAGTGACTTTGGCTTCCCCTCTGATAGTGAGAGGGAGGACAAGGGGGCCCATGGGCCCAGGCCAGA  140
U2OS-clone2-allele1 TGAACAGTGACTTTGGCTTCCCCTCTGATAGTGAGAGGGAGGACAAGGGGGCCCATGGGCCCAGGCCAGA  140
U2OS-clone2-allele2 TGAACAGTGACTTTGGCTTCCCCTCTGATAGTGAGAGGGAGGACAAGGGGGCCCATGGGCCCAGGCCAGA  140
```

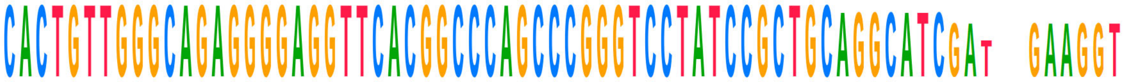

```
Consensus           CACTGTTGGGCAGAGGGGAGGTTCACGGCCCAGCCCGGGTCCTATCCGCTGCAGGCATCGA---GAAGGT
                            150       160       170       180       190       200       210
CHRONO-CDS          CACTGTTGGGCAGAGGGGAGGTTCACGGCCCAGCCCGGGTCCTATCCGCTGCAGGCATCGATC-GAAGGT  209
U2OS-clone1-allele1 CACTGTTGGGCAGAGGGGAGGTTCACGGCCCAGCCCGGGTCCTATCCGCTGCAGGCATC-----GAAGGT  205
U2OS-clone1-allele2 CACTGTTGGGCAGAGGGGAGGTTCACGGCCCAGCCCGGGTCCTATCCGCTGCAGGCATCGATTCGAAGGT  210
U2OS-clone2-allele1 CACTGTTGGGCAGAGGGGAGGTTCACGGCCCAGCCCGGGTCCTATCCGCTGC------------------  192
U2OS-clone2-allele2 CACTGTTGGGCAGAGGGGAGGTTCACGGCCCAGCCCGGGTCCTATCCGCTGCAGGCATCGA---------  201
```

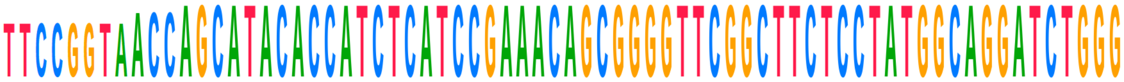

```
Consensus           TTCCGGTAACCAGCATACACCATCTCATCCGAAACAGCGGGGTTCGGCTTCTCCTATGGCAGGATCTGGG
                            220       230       240       250       260       270       280
CHRONO-CDS          TTCCGGTAACCAGCATACACCATCTCATCCGAAACAGCGGGGTTCGGCTTCTCCTATGGCAGGATCTGGG  279
U2OS-clone1-allele1 TTCCGGTAACCAGCATACACCATCTCATCCGAAACAGCGGGGTTCGGCTTCTCCTATGGCAGGATCTGGG  275
U2OS-clone1-allele2 TTCCGGTAACCAGCATACACCATCTCATCCGAAACAGCGGGGTTCGGCTTCTCCTATGGCAGGATCTGGG  280
U2OS-clone2-allele1 -----------AGCATACACCATCTCATCCGAAACAGCGGGGTTCGGCTTCTCCTATGGCAGGATCTGGG  251
U2OS-clone2-allele2 --------ACCAGCATACACCATCTCATCCGAAACAGCGGGGTTCGGCTTCTCCTATGGCAGGATCTGGG  263
```

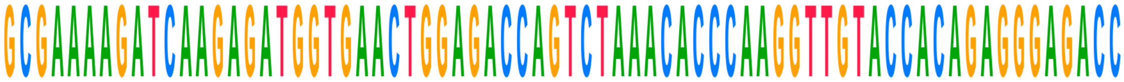

```
Consensus           GCGAAAAGATCAAGAGATGGTGAACTGGAGACCAGTCTAAACACCCAAGGTTGTACCACAGAGGGAGACC
                            290       300       310       320       330       340       350
CHRONO-CDS          GCGAAAAGATCAAGAGATGGTGAACTGGAGACCAGTCTAAACACCCAAGGTTGTACCACAGAGGGAGACC  349
U2OS-clone1-allele1 GCGAAAAGATCAAGAGATGGTGAACTGGAGACCAGTCTAAACACCCAAGGTTGTACCACAGAGGGAGACC  345
U2OS-clone1-allele2 GCGAAAAGATCAAGAGATGGTGAACTGGAGACCAGTCTAAACACCCAAGGTTGTACCACAGAGGGAGACC  350
U2OS-clone2-allele1 GCGAAAAGATCAAGAGATGGTGAACTGGAGACCAGTCTAAACACCCAAGGTTGTACCACAGAGGGAGACC  321
U2OS-clone2-allele2 GCGAAAAGATCAAGAGATGGTGAACTGGAGACCAGTCTAAACACCCAAGGTTGTACCACAGAGGGAGACC  333
```

Consensus	TGCTGTTTGCCCAGAAGTGTAAAGAACTCCAAGGATTTATACCTCCTCTCACAGACCTACTCAATGGGCT	
	360 370 380 390 400 410 420	
CHRONO-CDS	TGCTGTTTGCCCAGAAGTGTAAAGAACTCCAAGGATTTATACCTCCTCTCACAGACCTACTCAATGGGCT	419
U2OS-clone1-allele1	TGCTGTTTGCCCAGAAGTGTAAAGAACTCCAAGGATTTATACCTCCTCTCACAGACCTACTCAATGGGCT	415
U2OS-clone1-allele2	TGCTGTTTGCCCAGAAGTGTAAAGAACTCCAAGGATTTATACCTCCTCTCACAGACCTACTCAATGGGCT	420
U2OS-clone2-allele1	TGCTGTTTGCCCAGAAGTGTAAAGAACTCCAAGGATTTATACCTCCTCTCACAGACCTACTCAATGGGCT	391
U2OS-clone2-allele2	TGCTGTTTGCCCAGAAGTGTAAAGAACTCCAAGGATTTATACCTCCTCTCACAGACCTACTCAATGGGCT	403

Consensus	GAAGATGGGTCGTTTTGAGAGAGGATTAAGCAGTTTTCAGCAGAGTGTGGCAATGGACAGGATCCAGCGT	
	430 440 450 460 470 480 490	
CHRONO-CDS	GAAGATGGGTCGTTTTGAGAGAGGATTAAGCAGTTTTCAGCAGAGTGTGGCAATGGACAGGATCCAGCGT	489
U2OS-clone1-allele1	GAAGATGGGTCGTTTTGAGAGAGGATTAAGCAGTTTTCAGCAGAGTGTGGCAATGGACAGGATCCAGCGT	485
U2OS-clone1-allele2	GAAGATGGGTCGTTTTGAGAGAGGATTAAGCAGTTTTCAGCAGAGTGTGGCAATGGACAGGATCCAGCGT	490
U2OS-clone2-allele1	GAAGATGGGTCGTTTTGAGAGAGGATTAAGCAGTTTTCAGCAGAGTGTGGCAATGGACAGGATCCAGCGT	461
U2OS-clone2-allele2	GAAGATGGGTCGTTTTGAGAGAGGATTAAGCAGTTTTCAGCAGAGTGTGGCAATGGACAGGATCCAGCGT	473

Consensus	ATTGTAGGTGTTTTGCAGAAGCCACAGATGGGGGAACGTTACCTAGGAACCTTGCTACAGGTAGAAGGGA	
	500 510 520 530 540 550 560	
CHRONO-CDS	ATTGTAGGTGTTTTGCAGAAGCCACAGATGGGGGAACGTTACCTAGGAACCTTGCTACAGGTAGAAGGGA	559
U2OS-clone1-allele1	ATTGTAGGTGTTTTGCAGAAGCCACAGATGGGGGAACGTTACCTAGGAACCTTGCTACAGGTAGAAGGGA	555
U2OS-clone1-allele2	ATTGTAGGTGTTTTGCAGAAGCCACAGATGGGGGAACGTTACCTAGGAACCTTGCTACAGGTAGAAGGGA	560
U2OS-clone2-allele1	ATTGTAGGTGTTTTGCAGAAGCCACAGATGGGGGAACGTTACCTAGGAACCTTGCTACAGGTAGAAGGGA	531
U2OS-clone2-allele2	ATTGTAGGTGTTTTGCAGAAGCCACAGATGGGGGAACGTTACCTAGGAACCTTGCTACAGGTAGAAGGGA	543

Consensus	TGTTAAAGACTTGGTTTCCACAAATAGCTGCCCAGAAGTCATCATTGGGTGGTGGCAAGCATCAGCTGAC	
	570 580 590 600 610 620 630	
CHRONO-CDS	TGTTAAAGACTTGGTTTCCACAAATAGCTGCCCAGAAGTCATCATTGGGTGGTGGCAAGCATCAGCTGAC	629
U2OS-clone1-allele1	TGTTAAAGACTTGGTTTCCACAAATAGCTGCCCAGAAGTCATCATTGGGTGGTGGCAAGCATCAGCTGAC	625
U2OS-clone1-allele2	TGTTAAAGACTTGGTTTCCACAAATAGCTGCCCAGAAGTCATCATTGGGTGGTGGCAAGCATCAGCTGAC	630
U2OS-clone2-allele1	TGTTAAAGACTTGGTTTCCACAAATAGCTGCCCAGAAGTCATCATTGGGTGGTGGCAAGCATCAGCTGAC	601
U2OS-clone2-allele2	TGTTAAAGACTTGGTTTCCACAAATAGCTGCCCAGAAGTCATCATTGGGTGGTGGCAAGCATCAGCTGAC	613

Consensus	CAAGCATTTTCCAAGCCACCACAGTGATTCAGCTGCTTCCTCTCCTGCATCTCCTATGGAAAAGATGGAC	
	640 650 660 670 680 690 700	
CHRONO-CDS	CAAGCATTTTCCAAGCCACCACAGTGATTCAGCTGCTTCCTCTCCTGCATCTCCTATGGAAAAGATGGAC	699
U2OS-clone1-allele1	CAAGCATTTTCCAAGCCACCACAGTGATTCAGCTGCTTCCTCTCCTGCATCTCCTATGGAAAAGATGGAC	695
U2OS-clone1-allele2	CAAGCATTTTCCAAGCCACCACAGTGATTCAGCTGCTTCCTCTCCTGCATCTCCTATGGAAAAGATGGAC	700
U2OS-clone2-allele1	CAAGCATTTTCCAAGCCACCACAGTGATTCAGCTGCTTCCTCTCCTGCATCTCCTATGGAAAAGATGGAC	671
U2OS-clone2-allele2	CAAGCATTTTCCAAGCCACCACAGTGATTCAGCTGCTTCCTCTCCTGCATCTCCTATGGAAAAGATGGAC	683

```
Consensus            CAGACACAGCTAGGACATCTAGCTTTAAAACCAAAGCAGCCTTGGCACCTCACACAATGGCCAGCTATGA
                              710       720       730       740       750       760       770
CHRONO-CDS           CAGACACAGCTAGGACATCTAGCTTTAAAACCAAAGCAGCCTTGGCACCTCACACAATGGCCAGCTATGA  769
U2OS-clone1-allele1  CAGACACAGCTAGGACATCTAGCTTTAAAACCAAAGCAGCCTTGGCACCTCACACAATGGCCAGCTATGA  765
U2OS-clone1-allele2  CAGACACAGCTAGGACATCTAGCTTTAAAACCAAAGCAGCCTTGGCACCTCACACAATGGCCAGCTATGA  770
U2OS-clone2-allele1  CAGACACAGCTAGGACATCTAGCTTTAAAACCAAAGCAGCCTTGGCACCTCACACAATGGCCAGCTATGA  741
U2OS-clone2-allele2  CAGACACAGCTAGGACATCTAGCTTTAAAACCAAAGCAGCCTTGGCACCTCACACAATGGCCAGCTATGA  753
```

```
Consensus            ACCTCACCTGGATCCACACCACTCCAATTTGCAACCCCCCTCTCAGCTCCCCAGGTACTATCTCCTTTAG
                              780       790       800       810       820       830       840
CHRONO-CDS           ACCTCACCTGGATCCACACCACTCCAATTTGCAACCCCCCTCTCAGCTCCCCAGGTACTATCTCCTTTAG  839
U2OS-clone1-allele1  ACCTCACCTGGATCCACACCACTCCAATTTGCAACCCCCCTCTCAGCTCCCCAGGTACTATCTCCTTTAG  835
U2OS-clone1-allele2  ACCTCACCTGGATCCACACCACTCCAATTTGCAACCCCCCTCTCAGCTCCCCAGGTACTATCTCCTTTAG  840
U2OS-clone2-allele1  ACCTCACCTGGATCCACACCACTCCAATTTGCAACCCCCCTCTCAGCTCCCCAGGTACTATCTCCTTTAG  811
U2OS-clone2-allele2  ACCTCACCTGGATCCACACCACTCCAATTTGCAACCCCCCTCTCAGCTCCCCAGGTACTATCTCCTTTAG  823
```

```
Consensus            CCATGGTCCTTTAGGCACTGGAACCGGCATTGGCGTCATTCTTTTCCTCCAGCATGGAGTGCAACCCTTC
                              850       860       870       880       890       900       910
CHRONO-CDS           CCATGGTCCTTTAGGCACTGGAACCGGCATTGGCGTCATTCTTTTCCTCCAGCATGGAGTGCAACCCTTC  909
U2OS-clone1-allele1  CCATGGTCCTTTAGGCACTGGAACCGGCATTGGCGTCATTCTTTTCCTCCAGCATGGAGTGCAACCCTTC  905
U2OS-clone1-allele2  CCATGGTCCTTTAGGCACTGGAACCGGCATTGGCGTCATTCTTTTCCTCCAGCATGGAGTGCAACCCTTC  910
U2OS-clone2-allele1  CCATGGTCCTTTAGGCACTGGAACCGGCATTGGCGTCATTCTTTTCCTCCAGCATGGAGTGCAACCCTTC  881
U2OS-clone2-allele2  CCATGGTCCTTTAGGCACTGGAACCGGCATTGGCGTCATTCTTTTCCTCCAGCATGGAGTGCAACCCTTC  893
```

```
Consensus            ACCCACTCTGCCCCAACCACCCCAGTCCCACCTACTACAGCATCTCCTGTCATCCCTGGTGAGCCTATGA
                              920       930       940       950       960       970       980
CHRONO-CDS           ACCCACTCTGCCCCAACCACCCCAGTCCCACCTACTACAGCATCTCCTGTCATCCCTGGTGAGCCTATGA  979
U2OS-clone1-allele1  ACCCACTCTGCCCCAACCACCCCAGTCCCACCTACTACAGCATCTCCTGTCATCCCTGGTGAGCCTATGA  975
U2OS-clone1-allele2  ACCCACTCTGCCCCAACCACCCCAGTCCCACCTACTACAGCATCTCCTGTCATCCCTGGTGAGCCTATGA  980
U2OS-clone2-allele1  ACCCACTCTGCCCCAACCACCCCAGTCCCACCTACTACAGCATCTCCTGTCATCCCTGGTGAGCCTATGA  951
U2OS-clone2-allele2  ACCCACTCTGCCCCAACCACCCCAGTCCCACCTACTACAGCATCTCCTGTCATCCCTGGTGAGCCTATGA  963
```

```
Consensus            AACTATCTGGAGAGGGTCCTCGTTGCTACAGTTTGCCAGTAACTCTGCCATCAGACTGGAGCTATACCCT
                              990      1000      1010      1020      1030      1040      1050
CHRONO-CDS           AACTATCTGGAGAGGGTCCTCGTTGCTACAGTTTGCCAGTAACTCTGCCATCAGACTGGAGCTATACCCT  1049
U2OS-clone1-allele1  AACTATCTGGAGAGGGTCCTCGTTGCTACAGTTTGCCAGTAACTCTGCCATCAGACTGGAGCTATACCCT  1045
U2OS-clone1-allele2  AACTATCTGGAGAGGGTCCTCGTTGCTACAGTTTGCCAGTAACTCTGCCATCAGACTGGAGCTATACCCT  1050
U2OS-clone2-allele1  AACTATCTGGAGAGGGTCCTCGTTGCTACAGTTTGCCAGTAACTCTGCCATCAGACTGGAGCTATACCCT  1021
U2OS-clone2-allele2  AACTATCTGGAGAGGGTCCTCGTTGCTACAGTTTGCCAGTAACTCTGCCATCAGACTGGAGCTATACCCT  1033
```

ATCCCCTCCCAGTCTACCCACCTTGGCCAGAAAGATGACCATAGGACACCGGGAGCAGCAGAGAAGCCAT

1060 1070 1080 1090 1100 1110 1120

**Consensus**	ATCCCCTCCCAGTCTACCCACCTTGGCCAGAAAGATGACCATAGGACACCGGGAGCAGCAGAGAAGCCAT	
CHRONO-CDS	ATCCCCTCCCAGTCTACCCACCTTGGCCAGAAAGATGACCATAGGACACCGGGAGCAGCAGAGAAGCCAT	1119
U2OS-clone1-allele1	ATCCCCTCCCAGTCTACCCACCTTGGCCAGAAAGATGACCATAGGACACCGGGAGCAGCAGAGAAGCCAT	1115
U2OS-clone1-allele2	ATCCCCTCCCAGTCTACCCACCTTGGCCAGAAAGATGACCATAGGACACCGGGAGCAGCAGAGAAGCCAT	1120
U2OS-clone2-allele1	ATCCCCTCCCAGTCTACCCACCTTGGCCAGAAAGATGACCATAGGACACCGGGAGCAGCAGAGAAGCCAT	1091
U2OS-clone2-allele2	ATCCCCTCCCAGTCTACCCACCTTGGCCAGAAAGATGACCATAGGACACCGGGAGCAGCAGAGAAGCCAT	1103

CCTCCAGTTGCTGCTGATGCTCATCTTCTCAACCTCTAG

1130 1140 1150

**Consensus**	CCTCCAGTTGCTGCTGATGCTCATCTTCTCAACCTCTAG	
CHRONO-CDS	CCTCCAGTTGCTGCTGATGCTCATCTTCTCAACCTCTAG	1158
U2OS-clone1-allele1	CCTCCAGTTGCTGCTGATGCTCATCTTCTCAACCTCTAG	1154
U2OS-clone1-allele2	CCTCCAGTTGCTGCTGATGCTCATCTTCTCAACCTCTAG	1159
U2OS-clone2-allele1	CCTCCAGTTGCTGCTGATGCTCATCTTCTCAACCTCTAG	1130
U2OS-clone2-allele2	CCTCCAGTTGCTGCTGATGCTCATCTTCTCAACCTCTAG	1142

```
Consensus           MDSPSSVSSYSSYSLSSSFPTSPVNSDFGFPSDSEREDKGAHGPRPDTVGQRGGSRPSPGPIRCRHRXXX
                             10        20        30        40        50        60        70
CHRONO              MDSPSSVSSYSSYSLSSSFPTSPVNSDFGFPSDSEREDKGAHGPRPDTVGQRGGSRPSPGPIRCRHRSKV  70
U2OS-clone1-allele1 MDSPSSVSSYSSYSLSSSFPTSPVNSDFGFPSDSEREDKGAHGPRPDTVGQRGGSRPSPGPIRCRHRRFP  70
U2OS-clone1-allele2 MDSPSSVSSYSSYSLSSSFPTSPVNSDFGFPSDSEREDKGAHGPRPDTVGQRGGSRPSPGPIRCRHRFEG  70
U2OS-clone2-allele1 MDSPSSVSSYSSYSLSSSFPTSPVNSDFGFPSDSEREDKGAHGPRPDTVGQRGGSRPSPGPIRCS-----  65
U2OS-clone2-allele2 MDSPSSVSSYSSYSLSSSFPTSPVNSDFGFPSDSEREDKGAHGPRPDTVGQRGGSRPSPGPIRCRHR---  67

Consensus           XXXIHHLIR----NSGVRLLLWQDLGRKD------------------------QEMVNWRPV*-------
                             80        90       100       110       120       130       140
CHRONO              SGNQHTPSHPKQRGSASPMAGSGAKRSRDGELETSLNTQGCTTEGDLLFAQKCKELQGFIPPLTDLLNGL  140
U2OS-clone1-allele1 VTSIHHLIR----NSGVRLLLWQDLGRKD------------------------QEMVNWRPV*-------  105
U2OS-clone1-allele2 FR*-------------------------------------------------------------------  73
U2OS-clone2-allele1 ---IHHLIR----NSGVRLLLWQDLGRKD------------------------QEMVNWRPV*-------  97
U2OS-clone2-allele2 -TSIHHLIR----NSGVRLLLWQDLGRKD------------------------QEMVNWRPV*-------  101

Consensus           ----------------------------------------------------------------------
                            150       160       170       180       190       200       210
CHRONO              KMGRFERGLSSFQQSVAMDRIQRIVGVLQKPQMGERYLGTLLQVEGMLKTWFPQIAAQKSSLGGGKHQLT  210
U2OS-clone1-allele1 ----------------------------------------------------------------------  105
U2OS-clone1-allele2 ----------------------------------------------------------------------  73
U2OS-clone2-allele1 ----------------------------------------------------------------------  97
U2OS-clone2-allele2 ----------------------------------------------------------------------  101

Consensus           ----------------------------------------------------------------------
                            220       230       240       250       260       270       280
CHRONO              KHFPSHHSDSAASSPASPMEKMDQTQLGHLALKPKQPWHLTQWPAMNLTWIHTTPICNPPLSSPGTISFS  280
U2OS-clone1-allele1 ----------------------------------------------------------------------  105
U2OS-clone1-allele2 ----------------------------------------------------------------------  73
U2OS-clone2-allele1 ----------------------------------------------------------------------  97
U2OS-clone2-allele2 ----------------------------------------------------------------------  101

Consensus           ----------------------------------------------------------------------
                            290       300       310       320       330       340       350
CHRONO              HGPLGTGTGIGVILFLQHGVQPFTHSAPTTPVPPTTASPVIPGEPMKLSGEGPRCYSLPVTLPSDWSYTL  350
U2OS-clone1-allele1 ----------------------------------------------------------------------  105
U2OS-clone1-allele2 ----------------------------------------------------------------------  73
U2OS-clone2-allele1 ----------------------------------------------------------------------  97
U2OS-clone2-allele2 ----------------------------------------------------------------------  101

Consensus           ------------------------------------
                            360       370       380
CHRONO              SPPSLPTLARKMTIGHREQQRSHPPVAADAHLLNL*  386
U2OS-clone1-allele1 ------------------------------------  105
U2OS-clone1-allele2 ------------------------------------  73
U2OS-clone2-allele1 ------------------------------------  97
U2OS-clone2-allele2 ------------------------------------  101
```

Table S5. siRNA lists for knocking down nuclear transporters

siRNA name	Sequnence 5' to 3'
siKPNA5-205	GCUAACAGCAACACAGAAATT
	UUUCUGUGUUGCUGUUAGCTT
siKPNA5-424	CGAUGGACCCAAUGAUAAATT
	UUUAUCAUUGGGUCCAUCGTT
siKPNA5-973	GCGUAGACGAAGAGAAGAATT
	UUCUUCUCUUCGUCUACGCTT
siTNPO2-1680	GGAAAUACCAGCACAAGAATT
	UUCUUGUGCUGGUAUUUCCTT
siTNPO2-2334	UCAACAACCUGGUGGAAAUTT
	AUUUCCACCAGGUUGUUGATT
siTNPO2-1011	GGAUGAAGUACUCGGAAAUTT
	AUUUCCGAGUACUUCAUCCTT
siKPNB2-134	GUGGAAACCUGACGAGCAATT
	UUGCUCGUCAGGUUUCCACTT
siKPNB2-233	GGAACAACUUAAUCAGUAUTT
	AUACUGAUUAAGUUGUUCCTT
siKPNB2-578	UCAGAAGAUUUGUGAAGAUTT
	AUCUUCACAAAUCUUCUGATT
siCSE1L-2040	GGAAAGAACAGGAAAUAUUTT
	AAUAUUUCCUGUUCUUUCCTT
siCSE1-345	GAGAAUUGUUGAAGAUGAATT
	UUCAUCUUCAACAAUUCUCTT
siCSE1-1803	CUACAUGUUUGAAGCAAUATT
	UAUUGCUUCAAACAUGUAGTT
siIPO13-3213	GGGAAAGGUGGUACAGGAATT
	UUCCUGUACCACCUUUCCCTT
siIPO13-855	CGACAAGGUACCAGAGAUCTT
	GAUCUCUGGUACCUUGUCGTT
siIPO13-1742	GGUACACACUGCAGGAUGATT
	UCAUCCUGCAGUGUGUACCTT
siCtrl	UUCUCCGAACGUGUCACGUTT
	ACGUGACACGUUCGGAGAATT
